# Hierarchically porous waste human hair/chitosan composite mats enabling reusable capture of fresh and spent motor oils from freshwater and marine environments

**DOI:** 10.1039/d6ra04911g

**Published:** 2026-08-12

**Authors:** Van Dat Doan, Thi Kim Thuy Nguyen, Vy Anh Tran, Ngoc Nga Ho, Hien Y Hoang, Quoc Bao Tran, Van Thuan Le

**Affiliations:** a Faculty of Chemical Engineering, Industrial University of Ho Chi Minh City Ho Chi Minh City 700000 Vietnam; b Faculty of Environmental and Natural Sciences, Duy Tan University 03 Quang Trung Da Nang City 550000 Vietnam; c Faculty of Applied Science and Technology, Nguyen Tat Thanh University Ho Chi Minh City 700000 Vietnam; d Center for Advanced Chemistry, Institute of Research & Development, Duy Tan University 03 Quang Trung Da Nang City 550000 Vietnam levanthuan3@duytan.edu.vn; e Faculty of Environment, Ho Chi Minh City University of Natural Resources and Environment Ho Chi Minh City 70000 Vietnam tqbao@hcmunre.edu.vn

## Abstract

Human hair (HH) waste represents an abundant keratin-rich biopolymer with considerable potential for sustainable environmental remediation. In this study, highly porous and reusable oil sorbent mats were fabricated by integrating discarded HH fibers into a chitosan (CS) matrix through a facile sodium tripolyphosphate-mediated ionic crosslinking strategy. To optimize the composite architecture, a series of HH/CS mats with different HH/CS ratios were systematically prepared and evaluated in terms of porosity, wettability, mechanical properties, and oil sorption performance. All composite mats exhibited lightweight porous structures with porosities ranging from 78.5–81.8% and low bulk densities (0.262–0.302 g cm^−3^), enabling efficient flotation and oil capture on water surfaces. Among the investigated compositions, HH/CS-2.0 exhibited the best balance between structural integrity and adsorption performance, achieving the highest Young's modulus (146.79 MPa) and a maximum motor oil sorption capacity under bulk oil conditions of 4864.46 mg g^−1^, corresponding to nearly five times its own weight. The optimized mat demonstrated efficient removal of both fresh and used engine oils from freshwater and seawater surfaces. More importantly, it retained approximately 90% of its initial sorption capacity with less than 9% structural mass loss after five consecutive adsorption–desorption cycles, highlighting its excellent durability and regeneration potential. Post-reuse SEM, FTIR, and XRD analyses further confirmed the preservation of the composite framework after repeated operation. Comprehensive FTIR, XPS, and GC-MS investigations revealed that oil uptake was predominantly governed by physical mechanisms, including hydrophobic affinity, capillary-driven infiltration, and confinement within interconnected hierarchical pores, without inducing significant chemical transformation of either the sorbent matrix or the captured hydrocarbons. This work demonstrates a simple, low-cost, and scalable route for valorizing waste HH into high-performance bio-based sorbents with promising potential for oil spill remediation and oily wastewater treatment.

## Abstract 2

A hierarchically porous human hair/chitosan composite mat enables efficient and reusable capture of fresh and spent motor oils from freshwater and marine environments through capillary-driven physical confinement. Image creation assisted by Google Gemini.

## Graphical abstract

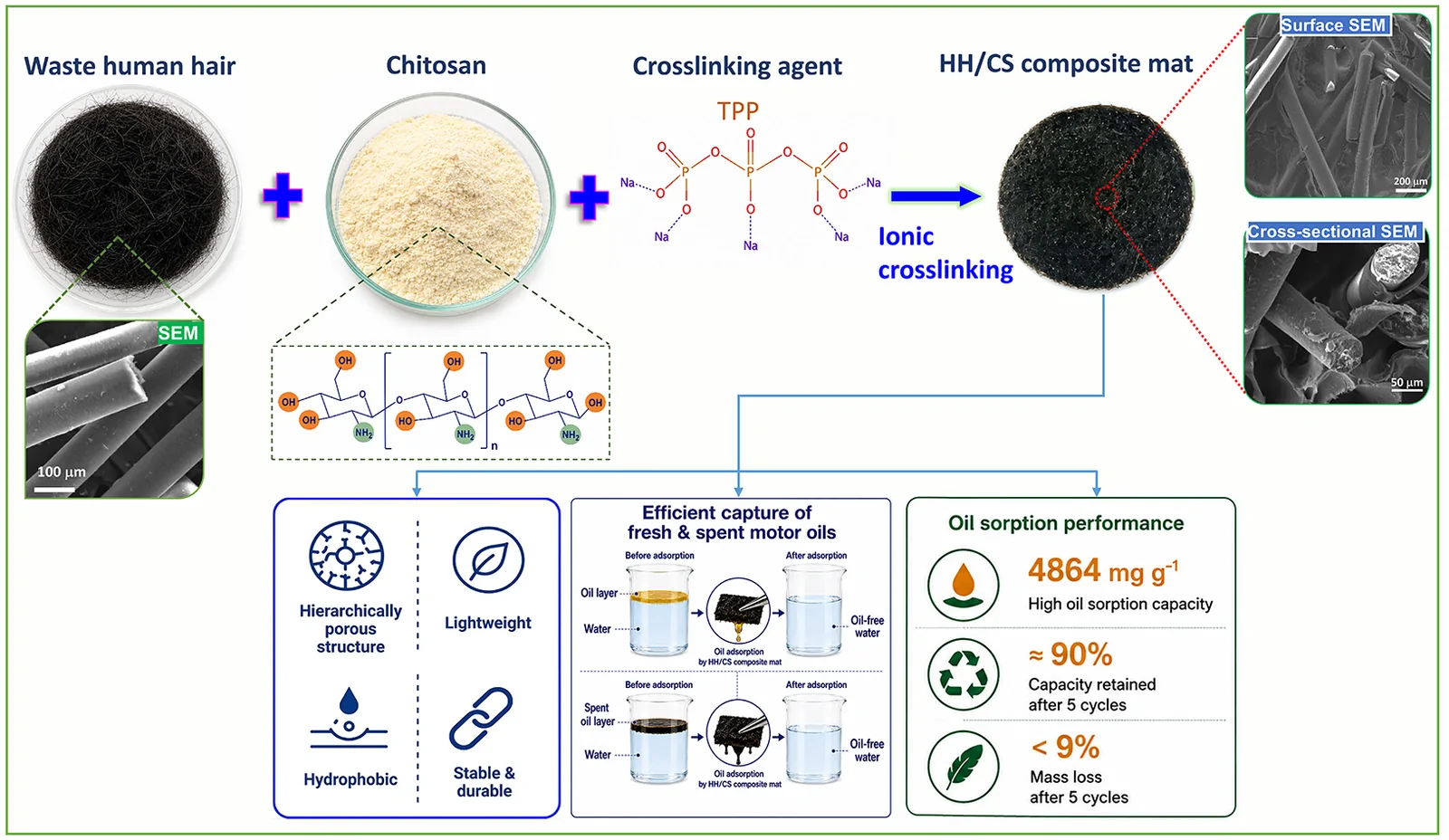

## 1. Introduction

Oil contamination originating from industrial discharge, transportation accidents, petroleum leakage, and improper disposal of lubricating oils has become one of the most persistent environmental challenges threatening aquatic ecosystems and human health. In particular, the release of engine lubricating oils into freshwater and marine environments can severely deteriorate water quality due to their complex composition, high persistence, and toxicity toward aquatic organisms.^1^ Waste engine oils are especially problematic because they contain not only degraded hydrocarbons but also combustion-derived residues, heavy metals, oxidized compounds, and various toxic additives generated during engine operation.^2^ Therefore, the development of efficient, low-cost, and environmentally sustainable materials for oil spill remediation and oily wastewater treatment remains an urgent global priority.

Numerous physical, chemical, and biological remediation approaches have been proposed for oil removal, including *in situ* burning, chemical dispersants, flotation, membrane separation, biodegradation, and adsorption-based technologies.^3^ Among these strategies, adsorption has attracted considerable attention owing to its operational simplicity, high efficiency, facile recovery process, and adaptability to diverse environmental conditions.^4,5^ Conventional commercial sorbents, such as polypropylene-based materials, generally exhibit excellent oil uptake capacity; however, their petroleum-derived origin, poor biodegradability, high production cost, and generation of secondary plastic waste significantly limit their long-term environmental sustainability.^6,7^ Consequently, increasing efforts have been devoted toward the exploration of renewable and biodegradable bio-based sorbents derived from agricultural residues, lignocellulosic biomass, natural fibers, and biopolymers.^4,8,9^

Human hair (HH), an abundant keratin-rich biowaste generated continuously from barbershops and salons, has emerged as a promising candidate for oil sorption applications. Compared with many other keratin-containing wastes such as feathers, wool, horns, or animal hairs, waste HH is readily available in urban areas, can be collected without seasonal or agricultural dependence, requires no animal processing, and is generally discarded without beneficial utilization. These characteristics make HH an attractive feedstock for sustainable resource recovery within a circular economy framework. Owing to its intrinsic hydrophobicity, rough surface morphology, hollow microstructure, and excellent chemical stability, HH possesses a natural affinity toward hydrocarbon compounds and has long been recognized for its ability to adsorb oils and organic pollutants.^10^ The fibrous morphology and high sulfur-containing keratin content further contribute to its remarkable resistance against chemical degradation in aqueous environments. Previous studies have demonstrated the feasibility of employing waste HH for oil spill remediation. For example, Jilagam *et al.* reported that raw HH could effectively remove diesel oil from synthetic seawater,^11^ while Erpınar *et al.* demonstrated the potential of incorporating waste HH into natural felt mats for marine oil adsorption.^12^ These findings collectively demonstrate the considerable potential of hair-derived materials as sustainable sorbents for oil spill cleanup. Nevertheless, pristine HH fibers still suffer from several practical limitations, including poor structural cohesion, limited mechanical integrity, irregular packing density, and difficulties in handling and recovery during repeated sorption–desorption operations. Such limitations may restrict oil transport efficiency and reduce the long-term operational stability of loose fibrous sorbents in real aquatic environments.

Chitosan (CS), a biodegradable polysaccharide derived from chitin deacetylation, has also been extensively investigated as an environmentally benign adsorbent because of its non-toxicity, biocompatibility, film-forming ability, and abundant functional groups.^13^ The presence of amino and hydroxyl groups enables facile chemical modification and structural tailoring of CS-based materials into membranes, sponges, hydrogels, aerogels, and porous scaffolds for pollutant removal. However, pristine CS materials commonly exhibit poor mechanical stability and structural collapse under prolonged aqueous exposure unless additional crosslinking or reinforcement strategies are introduced. Furthermore, although CS-based aerogels and nanostructured sorbents have demonstrated promising oil uptake performance, many reported fabrication methods require complicated synthesis routes, freeze-drying processes, hydrophobic surface modifications, or expensive nanomaterials, thereby limiting their scalability and economic feasibility.^14^

To overcome these limitations, hybridization of hair-based materials with CS presents an attractive strategy for constructing mechanically stable and highly porous sorption systems. Although recent studies have explored hair–CS combinations, such as precipitating CS onto pulverized synthetic hair particles^10^ or mechanically blending human hair with sheep wool,^12^ these configurations still exhibit important structural limitations. The former relies on fragmented particulate assemblies that lack a continuous self-supporting porous network, resulting in limited structural robustness during repeated sorption–desorption operations. The latter is produced through traditional mechanical felting without a chemically crosslinked supporting framework, making the loose fibrous architecture more susceptible to irreversible deformation and gradual fiber disintegration during cyclic compression and reuse.

Beyond the issues of structural cohesion, processing scalability remains a critical bottleneck. Most previously reported CS-based frameworks or hybrid bio-based sorbents rely on energy-intensive lyophilization (freeze-drying) protocols or toxic chemical modifications (such as silane functionalization or fluorinated coatings) to prevent structural collapse and achieve satisfactory buoyancy.^15–17^ These fabrication strategies inevitably increase processing complexity, production cost, and environmental burden, thereby limiting their practical scalability. Therefore, there remains a need for a structurally integrated bio-based sorbent that combines facile fabrication, mechanical robustness, interconnected macroporous transport pathways, and efficient oil confinement without additional surface functionalization or energy-intensive processing.

To circumvent these processing and mechanical limitations, the present work introduces a structural engineering strategy in which discarded HH fibers are immobilized within a continuous TPP-crosslinked CS framework to construct a self-supporting, hierarchically porous composite mat under ambient conditions. In this architecture, CS functions not merely as a binder but as a mechanically integrated three-dimensional network that locks the high-aspect-ratio keratin fibers while preserving interconnected macroporous channels throughout the composite. This rational structural design simultaneously provides low bulk density, interconnected capillary pathways, improved mechanical integrity, facile handling and recovery, and efficient physical confinement of hydrocarbons without requiring freeze-drying, chemical hydrophobic functionalization, or nanofiller incorporation.

Compared with previously reported hair-based sorbents, CS-derived aerogels, and hybrid bio-based composites, the present HH/CS composite mat provides several distinctive features. It combines a lightweight floating hierarchically porous architecture with a simple ambient-temperature ionic crosslinking process while simultaneously enhancing structural integrity, interconnected capillary pathways, and handling performance without requiring freeze-drying, hydrophobic surface modification, or nanofiller incorporation.

Accordingly, this study aims to develop a sustainable, reusable, and highly porous HH/CS composite mat *via* a facile sodium TPP-mediated ionic crosslinking strategy for efficient oil spill remediation by valorizing waste human hair as a renewable reinforcing material. The effects of HH/CS blending ratio and key operating parameters (including contact time, sorbent dosage, temperature, oil volume, and mat geometry) on the oil sorption performance and reusability of the composite were systematically investigated. The structure–property–performance relationships governing the sorption behavior were further elucidated through comprehensive physicochemical characterization. The findings provide a simple, scalable, and environmentally benign strategy for converting keratin-rich HH waste into high-performance bio-based sorbents for oil spill cleanup and oily wastewater treatment.

## 2. Experimental

### 2.1 Materials

HH waste was collected from local barbershops in Da Nang. To better represent a realistic post-consumer waste stream, the collected hair was intentionally used as a mixed feedstock without sorting according to donor characteristics (*e.g.*, age or gender) or previous cosmetic treatments. Although such factors may introduce some variability in the physical properties of individual fibers, the use of mixed waste HH better reflects practical large-scale collection scenarios for waste valorization and circular economy applications. Prior to composite fabrication, the collected hair was thoroughly washed, dried, and cut into short fibers to minimize contamination and improve material uniformity. Commercial motorcycle engine oil (Idemitsu 4T SL/MA 10W-40) was purchased from a local supplier and used as the fresh oil sorbate without further purification. Used motorcycle engine oil was collected after routine motorcycle engine operation and filtered to remove large suspended impurities before use. CS (degree of deacetylation: ≥90.0%) was supplied by Yangzhou Rixing Bio-Tech Co., Ltd, China. Acetic acid (CH_3_COOH, ≥99%), sodium triphosphate pentabasic (Na_5_P_3_O_10_, ≥98.0%), *n*-hexane (≥99.0%), and hydrogen peroxide (H_2_O_2_, 30%) were of analytical grade and purchased from Sigma-Aldrich. Natural seawater was collected from My Khe Beach (Da Nang, Vietnam) at a distance of approximately 100 m from the shoreline (pH = 7.87, conductivity = 48.4 mS cm^−1^). Distilled water was used throughout the study.

### 2.2 Pretreatment of HH

The collected HH was manually cleaned to remove coarse impurities and washed with a 5% (v/v) commercial shampoo solution to eliminate grease and surface contaminants. After repeated rinsing with distilled water, the hair was dried at 60 °C and cut into short fibers (2–5 mm). To enhance surface cleanliness and interfacial compatibility with the CS matrix, the fibers were immersed in a 3% H_2_O_2_ solution for 30 min at room temperature. Finally, the treated fibers were rinsed thoroughly with distilled water until the pH of the rinse water reached approximately 7, and then dried at 60 °C before fabrication.

### 2.3 Fabrication of HH/CS mats

**Scheme 1:**
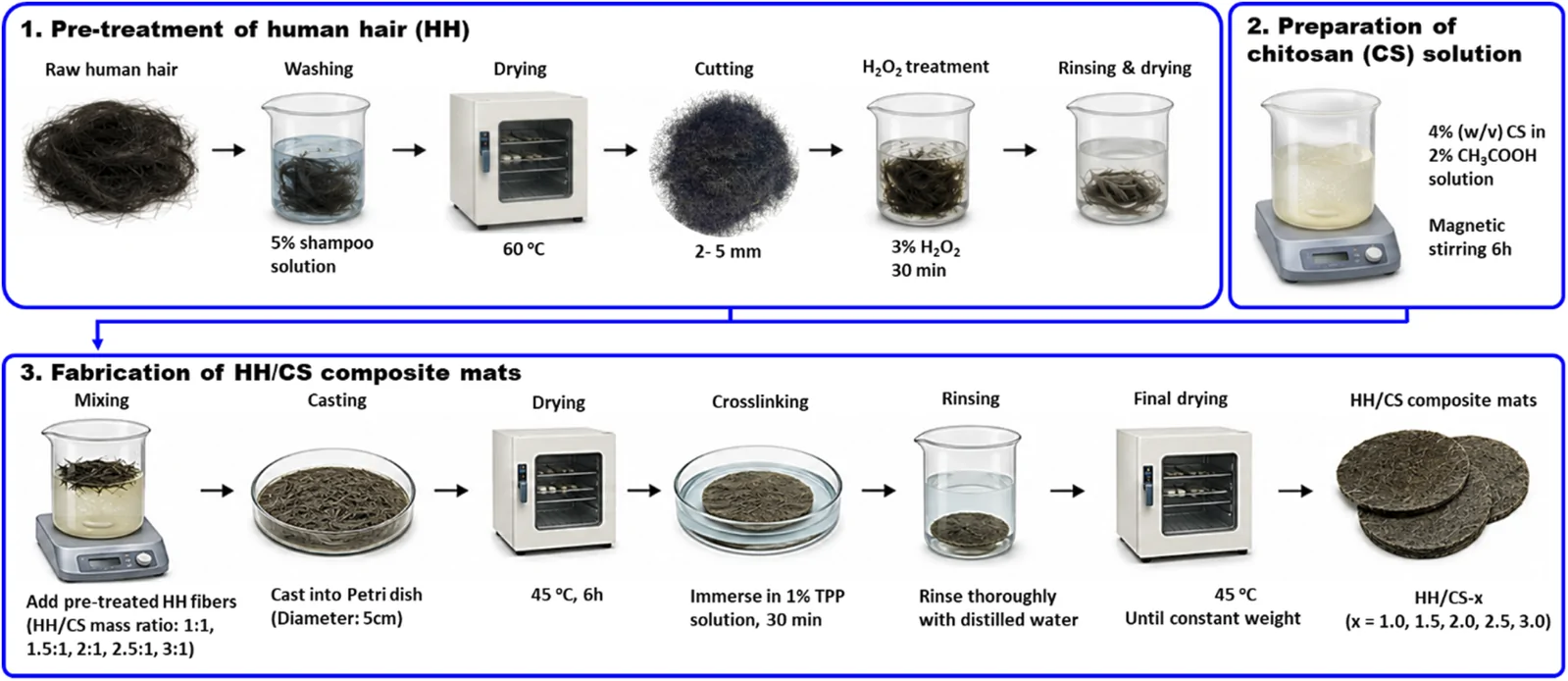
Schematic illustration of the synthesis process for HH/CS composite mats.

A 4% (w/v) CS solution was prepared by dissolving CS in 2% acetic acid solution under magnetic stirring for 6 h until a homogeneous viscous solution was obtained. The pretreated HH fibers were then incorporated into the CS solution at different mass ratios of HH/CS (1 : 1, 1.5 : 1, 2 : 1, 2.5 : 1, and 3 : 1). Specifically, 0.4, 0.6, 0.8, 1.0, and 1.2 g of HH fibers were added to 10 mL of the 4% CS solution, respectively. The resulting mixtures were mechanically stirred until uniform fiber dispersion was achieved. Subsequently, each mixture was cast into a Petri dish (diameter: 5 cm) and dried at 45 °C for 6 h to obtain preliminary mat structures. The formed mats were then immersed in 10 mL of 1% sodium tripolyphosphate solution for 30 min to induce ionic crosslinking within the CS matrix. After crosslinking, the composite mats were rinsed thoroughly with distilled water to remove residual reagents and dried again at 45 °C until constant weight (defined as a mass change of <1 mg between two consecutive weighings at 1 h intervals). The obtained samples were denoted as HH/CS-*x*, where *x* represents the HH-to-CS mass ratio (1.0, 1.5, 2.0, 2.5, and 3.0). The fabrication process of the HH/CS composite mats is illustrated in Scheme 1.

### 2.4 Characterization

The surface morphology and elemental distribution of the composite mats were examined using scanning electron microscopy equipped with energy-dispersive X-ray spectroscopy (SEM-EDX, TESCAN VEGA 3, Czech Republic). The crystalline structure of the samples was analyzed by X-ray diffraction (XRD, SmartLab, Rigaku, Japan) using Cu Kα radiation (*λ* = 1.5406 Å) operated in Bragg–Brentano geometry. The thermal stability and decomposition behavior were investigated using simultaneous thermal analysis (STA 6000, PerkinElmer, USA) over a temperature range of 30–700 °C at a heating rate of 10 °C min^−1^ under an air atmosphere. Fourier-transform infrared (FTIR) spectra were recorded using a Bruker Tensor 37 spectrometer (Germany) within the wavenumber range of 4000–400 cm^−1^ with 32 scans at a resolution of 2 cm^−1^ using the KBr pellet method. The surface chemical composition and chemical states of the constituent elements were analyzed by X-ray photoelectron spectroscopy (XPS, Nexsa X10, Thermo Fisher Scientific, USA) equipped with a monochromatic Al Kα X-ray source (*hν* = 1486.6 eV). High-resolution core-level spectra of C 1s, O 1s, N 1s, and S 2p were acquired at a pass energy of 50 eV with a step size of 0.1 eV, and all binding energies were calibrated against the adventitious C 1s peak at 284.8 eV. Mechanical properties, including tensile strength, Young's modulus, and elongation at break, were determined using a universal testing machine (UTM, YM-H4202-H02, Taiwan). Rectangular specimens (10 × 50 mm) were tested at a crosshead speed of 10 mm min^−1^. Five independent specimens were tested for each sample, and the reported values are presented as mean ± standard deviation. The diameter and thickness of the composite mats were measured using a digital vernier caliper (Mitutoyo, Japan; accuracy ± 0.01 mm). For each independently prepared mat, both the diameter and thickness were measured at three different locations, and the average values were recorded. Six independently prepared mats were characterized for each composition, and the reported values are presented as mean ± standard deviation. The surface wettability of the mats was evaluated by water contact angle (WCA) measurements using an optical contact angle system (DataPhysics OCA) with a 5 µL distilled water droplet at ambient temperature. Residual oil compositions during the adsorption tests were analyzed using gas chromatography-mass spectrometry (GC-MS, Agilent 7890A/5975C) after extraction with *n*-hexane.

### 2.5 Oil sorption experiments

Oil sorption experiments were conducted using both fresh and used motorcycle engine oils. Composite mats were cut into specimens of approximately 10 × 10 mm and dried prior to use. The initial dry weight of each sample (*m*_0_) was recorded before immersion in a 10 mL glass vial containing 5 mL of oil. After adsorption for a predetermined period, the samples were removed from the oil and allowed to drain for 2 min to eliminate excess unbound oil. The weight of the oil-loaded sample at adsorption time *t* (*m*_*t*_) was measured. The oil sorption capacity (*Q*) was calculated according to the following equation:1
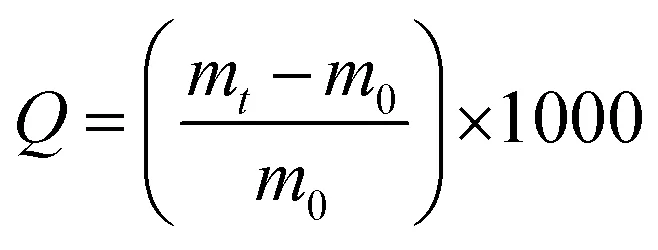
where *Q* (mg g^−1^) is the oil sorption capacity; *m*_0_ (g) is the initial dry mass of the composite mat; and *m*_*t*_ (g) is the mass of the oil-loaded mat at adsorption time *t*. The multiplication factor of 1000 converts the sorption capacity from g g^−1^ to mg g^−1^.

The effects of adsorption temperature, mat dimensions, mat thickness, and initial oil volume on the oil sorption performance were systematically investigated to evaluate the adsorption behavior of the prepared composite mats under different operating conditions.

### 2.6 Reusability and oil sorption on water surface

For reusability evaluation, the oil-loaded mats were desorbed by immersion in *n*-hexane for 15 min. The regenerated samples were subsequently dried and reused for five consecutive adsorption–desorption cycles. To simulate oil spill conditions, experiments were conducted using 50 mL of distilled water or natural seawater with 3 mL of oil floating on the surface. Composite mats (10 × 10 mm) were placed onto the oil layer for 30 min. To eliminate interference from water uptake by the CS matrix, the adsorbed oil was extracted by immersing the mats in 20 mL of *n*-hexane under constant stirring for 15 min. The resulting extract was transferred to a pre-weighed weighing bottle, and the solvent was evaporated at 40 °C in a vacuum oven until a constant weight was achieved. The net oil sorption capacity was then determined gravimetrically. The structural stability of the mats after repeated cycles was further characterized by FTIR, XRD, and SEM analyses. All oil sorption experiments were performed in triplicate using independently prepared composite mats under identical experimental conditions. The reported values are presented as mean ± standard deviation, and the corresponding error bars are shown in the figures where applicable.

## 3. Results and discussion

### 3.1. Effect of HH/CS ratio on structural, physical, and adsorption properties

#### 3.1.1 Formation and structural evolution of HH/CS composite mats

The structural organization and interfacial interactions within composite sorbent materials play important roles in determining their dimensional stability, porosity, wettability, and ultimately oil sorption performance. In the present study, HH fibers were incorporated into a CS matrix to construct a fibrous three-dimensional sorption network, while TPP was employed as an ionic crosslinking agent to improve the structural integrity of the resulting composite mats.

**Fig. 1:**
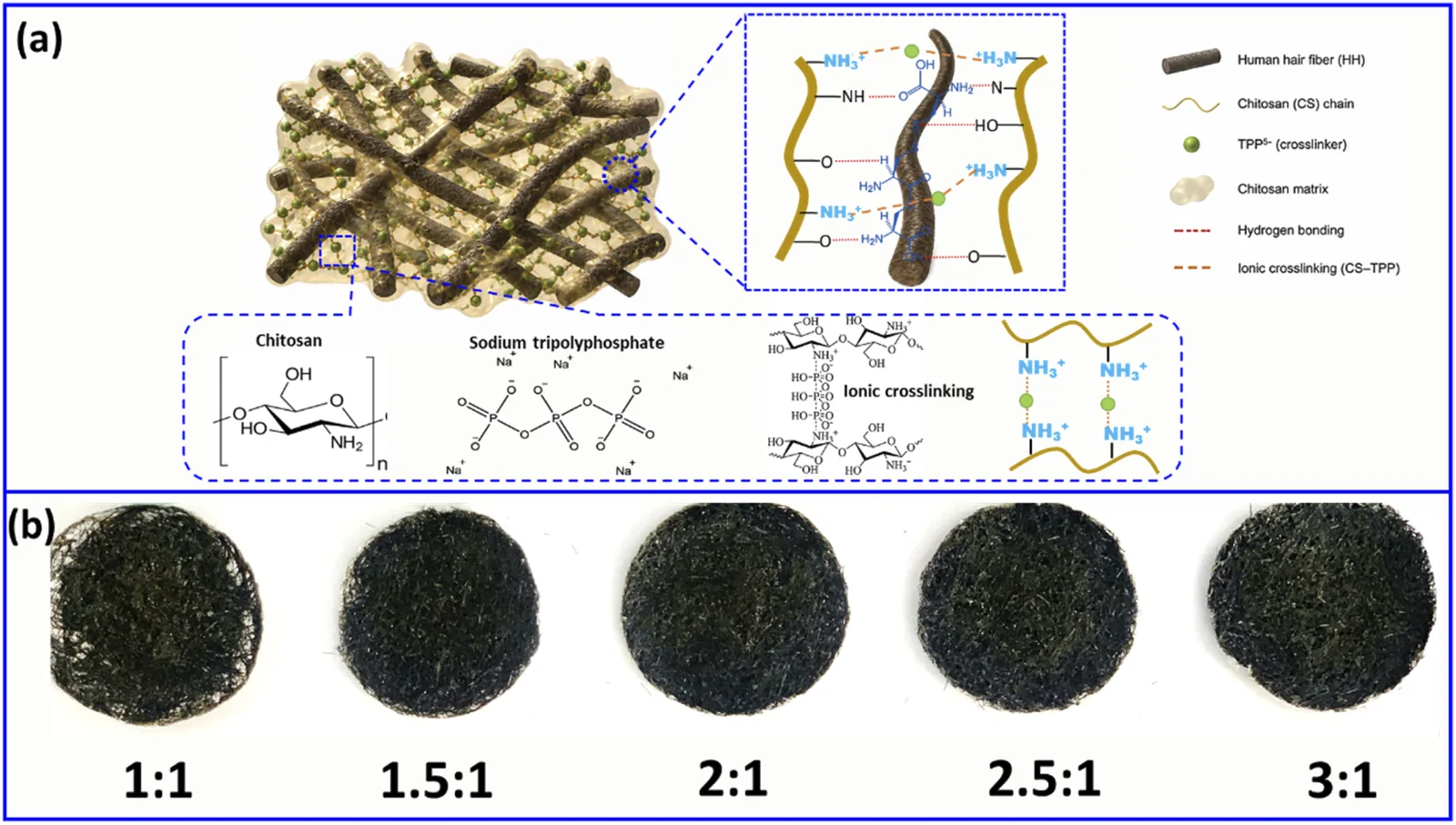
Schematic illustration of the internal structure and interaction mechanism of HH/CS composite mats crosslinked with TPP (a), and digital photographs of HH/CS composite mats prepared at different HH/CS mass ratios (b).

As schematically illustrated in Fig. 1a, the HH fibers were embedded within the CS matrix through hydrogen bonding and physical interfacial entanglement. The abundant hydroxyl, amino, and amide functional groups present in both keratin fibers and CS could facilitate intermolecular interactions at the HH–CS interface. In addition, TPP induced ionic crosslinking among protonated amino groups of CS, thereby stabilizing the interconnected network structure of the composite mats.

The HH/CS mass ratio is expected to strongly influence the structural characteristics of the prepared materials because the relative amount of fibrous reinforcement and polymeric binding phase directly affects matrix shrinkage, packing density, pore formation, and network compactness during the casting and drying processes. Therefore, different HH/CS mass ratios were investigated, and the resulting composite mats are shown in Fig. 1b. All samples maintained an integrated mat-like structure after casting, drying, and TPP crosslinking, indicating effective incorporation of HH fibers into the CS matrix.

#### 3.1.2 Dimensional characteristics and porosity

**Fig. 2:**
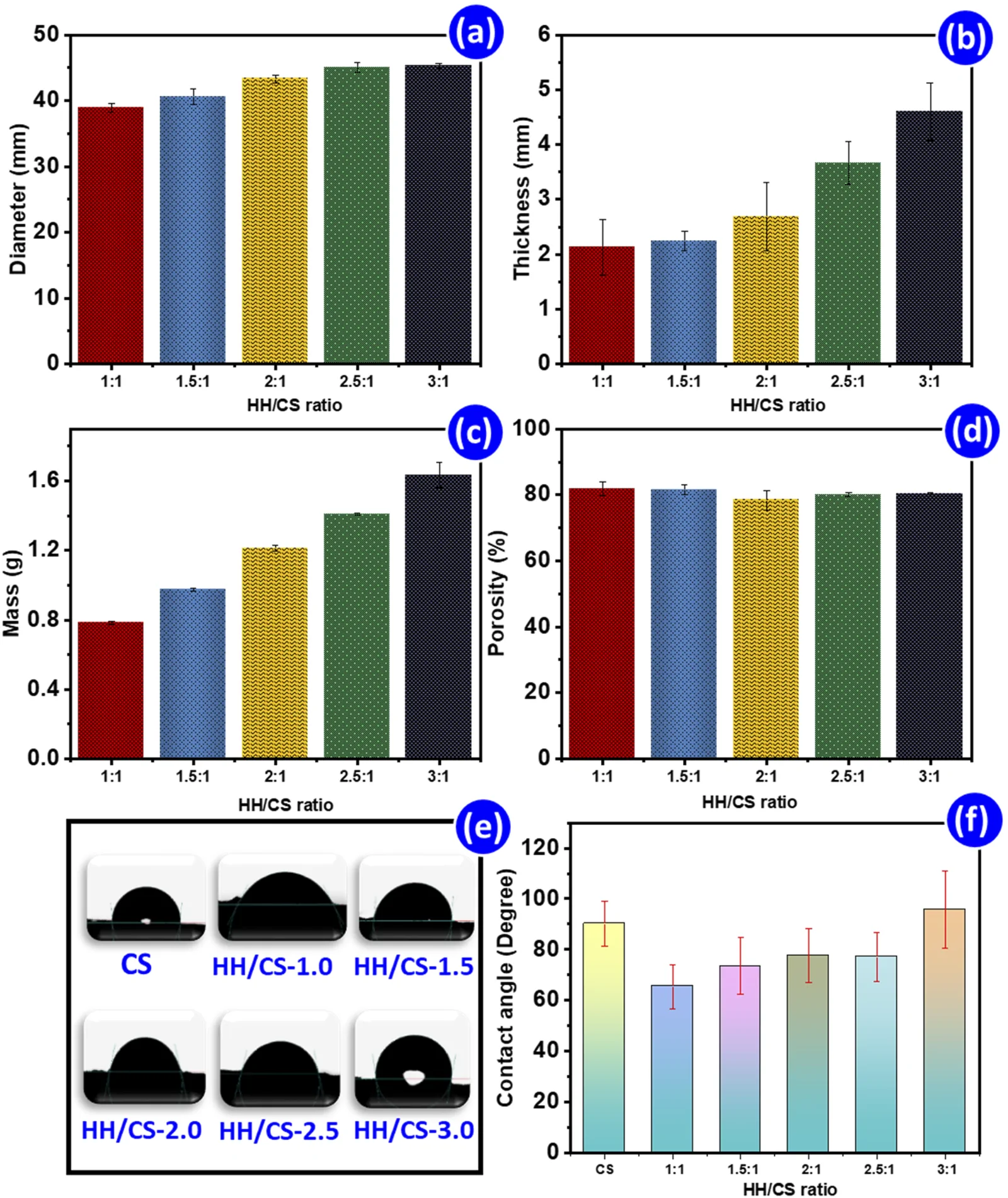
Physical characteristics and wettability properties of HH/CS composite mats prepared at different HH/CS mass ratios: (a) diameter, (b) thickness, (c) mass, and (d) porosity of the composite mats; (e) representative water contact angle images and (f) corresponding water contact angle values of pristine CS and HH/CS composite mats.

The physical characteristics of the prepared mats are summarized in Table S1 and Fig. 2a–c. Although all samples were initially cast in Petri dishes with a diameter of 5 cm, the final diameters of the obtained mats were noticeably smaller, suggesting structural shrinkage during solvent evaporation and drying of the CS matrix. Interestingly, increasing the HH content gradually reduced the shrinkage behavior, as evidenced by the increase in average mat diameter from 38.93 ± 0.69 mm for HH/CS-1.0 to 45.31 ± 0.34 mm for HH/CS-3.0. This phenomenon could be attributed to the presence of interconnected HH fibers, which acted as a physical supporting framework that restricted excessive contraction of the CS matrix during drying. A similar trend was observed for the thickness and mass of the composite mats. The average thickness increased from 2.14 ± 0.51 mm to 4.60 ± 0.53 mm with increasing HH loading, accompanied by a gradual increase in mat mass. This behavior suggests that higher HH loading produced a bulkier and less compact internal structure.

Porosity is a key structural parameter governing oil diffusion and storage within porous sorbents. The porosity values of the HH/CS composite mats were determined based on bulk and theoretical solid densities, as described in the SI, and the obtained results are presented in Table S2 and Fig. 2d.

All prepared composite mats exhibited high porosity values ranging from 78.5 to 81.8%, confirming the formation of highly porous three-dimensional structures after casting and TPP crosslinking. In addition, the bulk densities of all samples were remarkably low (0.262–0.302 g cm^−3^), which were substantially lower than the density of water. Such low-density characteristics are highly advantageous for oil spill remediation because they enable the composite mats to naturally float on water surfaces during the oil sorption process. The highly porous and lightweight structures could be attributed to the random interconnection of HH fibers within the CS matrix, which generated abundant void spaces and prevented excessive packing of the polymer chains during drying.

Interestingly, the porosity did not vary linearly with HH loading (Fig. 2d). The porosity decreased from 81.8% for HH/CS-1.0 to 78.5% for HH/CS-2.0, suggesting a more compact packing arrangement due to improved integration between the HH network and the CS matrix. Further increasing the HH content slightly increased the porosity to approximately 80%, likely because additional fiber entanglement generated more inter-fiber voids. Overall, the internal architecture of the HH/CS mats was governed by the balance between matrix shrinkage and fiber packing density. Although the mat thickness increased progressively with increasing HH loading (Fig. 2b), this geometric change did not directly result in higher porosity because the increase in thickness was accompanied by a corresponding increase in the mass of the composite mats, leading to only minor variations in bulk density.

#### 3.1.3 Wettability properties

The surface wettability of the HH/CS composite mats was evaluated by water contact angle (WCA) measurements to assess their potential for selective oil–water separation (Fig. 2e and f). The pristine CS mat exhibited a water contact angle of approximately 90.2°. Although CS contains abundant hydrophilic amino and hydroxyl groups, previous studies have shown that dried CS films may exhibit relatively hydrophobic behavior owing to molecular rearrangement and strong interchain hydrogen bonding during film formation, which reduce the exposure of polar groups at the surface.^18,19^ For porous fibrous materials, the measured WCA is an apparent value that is governed not only by the intrinsic surface chemistry but also by surface roughness and capillary infiltration into the porous structure.

Interestingly, the incorporation of HH fibers initially decreased the apparent water contact angle to 65.5° for HH/CS-1.0. This counterintuitive drop is attributed to capillary-driven wetting; low HH loading disrupted the compact CS matrix, creating localized surface voids and interconnected pores that drew water droplets inward *via* capillary action, while the hydrophobic hair cuticles remained largely masked beneath the CS layer.^20^ Similar capillary-assisted wetting phenomena have also been reported for other porous composite materials, where liquid infiltration into interconnected pore structures leads to a reduction in the apparent WCA.^21^ As the HH content increased further, the apparent WCA showed an overall increasing trend, ultimately reaching 95.7° for HH/CS-3.0. At higher HH loadings, the progressively exposed hydrophobic keratin-rich cuticles became the dominant surface feature. Under these conditions, the hierarchical fibrous roughness acted to amplify the intrinsic hydrophobicity of the exposed hair surfaces, resulting in a higher apparent WCA.^22^ Notably, the WCA of HH/CS-3.0 exceeded 90°, confirming its favorable hydrophobic nature required for selective oil sorption on water surfaces.

#### 3.1.4 Mechanical properties

**Fig. 3:**
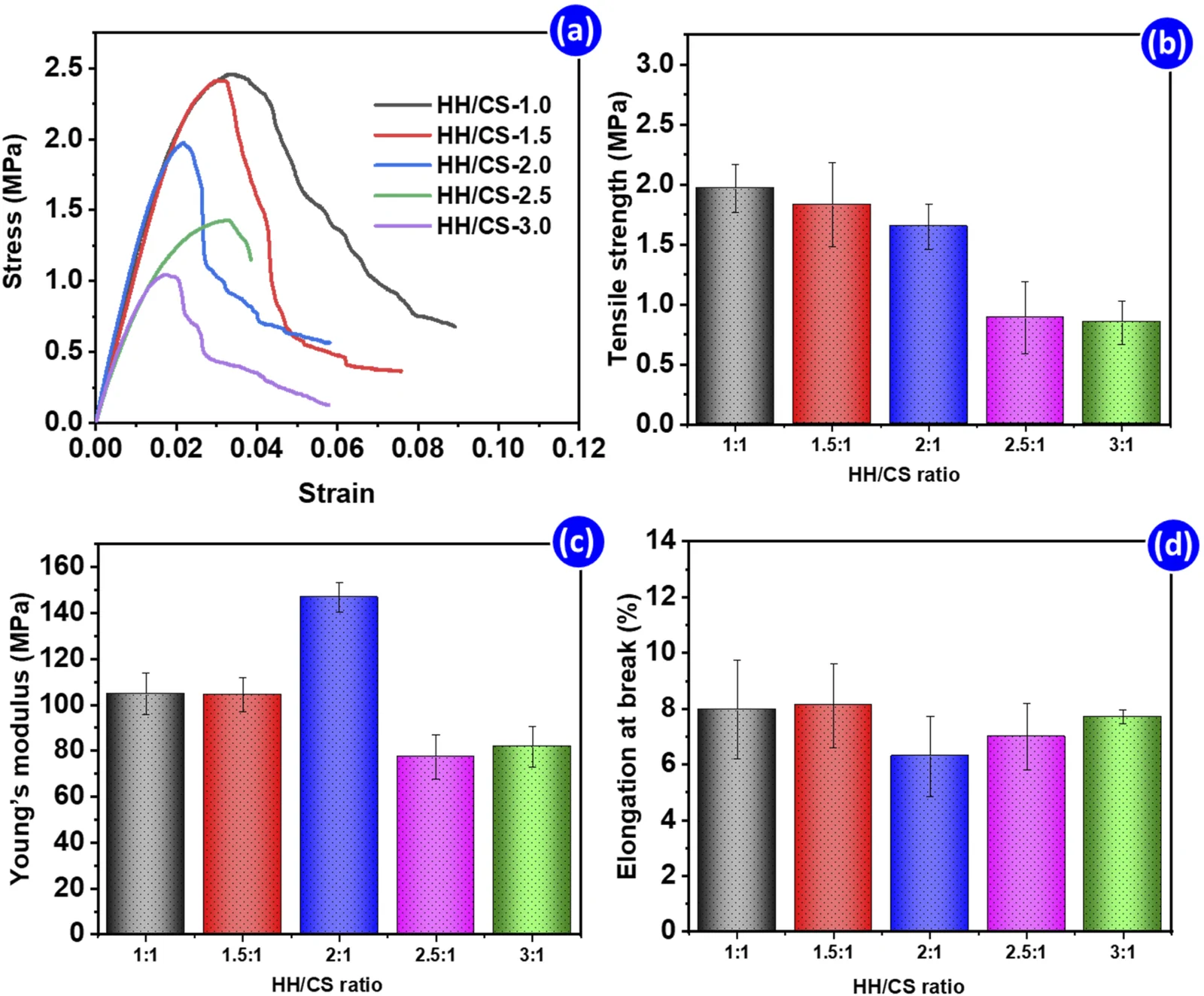
Mechanical properties of HH/CS composite mats prepared at different HH/CS mass ratios: (a) stress–strain curves, (b) tensile strength, (c) Young's modulus, and (d) elongation at break of the composite mats.

Mechanical stability is a critical requirement for oil-sorbent mats to withstand repetitive squeezing and cyclic recovery operations. Therefore, the tensile behaviors of the HH/CS composite mats were investigated, with stress–strain profiles and derived parameters summarized in Fig. 3.

As shown in Fig. 3a, all composite mats exhibited typical non-linear deformation behavior characteristic of porous fibrous polymer composites. The tensile strength gradually decreased from 1.97 MPa for HH/CS-1.0 to 0.85 MPa for HH/CS-3.0 (Fig. 3b). A similar decreasing trend was also observed for elongation at break at intermediate HH loading, although the values remained within a relatively narrow range of 6.29–8.11% (Fig. 3d). The reduction in tensile strength at high HH loading can be attributed to decreased continuity of the CS matrix and weaker interfacial stress transfer between adjacent fibers. At lower HH contents, the CS matrix effectively encapsulated the HH fibers, forming a cohesive network capable of distributing tensile stress more uniformly. In contrast, excessive HH incorporation disrupted matrix continuity and increased structural heterogeneity, thereby reducing the overall mechanical integrity of the mats.

Interestingly, the Young's modulus increased from approximately 104 MPa for HH/CS-1.0 and HH/CS-1.5 to 146.79 MPa for HH/CS-2.0 (Fig. 3c), indicating an optimal balance between the reinforcing effect of rigid keratin fibers and the binding capability of the CS matrix. Well-dispersed HH fibers likely restricted polymer chain mobility and improved stress transfer within the composite network, consistent with previous reports.^23^ Further increasing the HH content to HH/CS-2.5 and HH/CS-3.0 reduced the Young's modulus to 77.37 and 81.74 MPa, respectively. This decrease is attributed to insufficient CS matrix coverage, weaker interfacial adhesion, and the formation of thicker, more porous structures with lower structural compactness. Although such architectures are beneficial for oil sorption, excessive HH loading compromises the mechanical cohesion of the three-dimensional network. The stress–strain curves further revealed that HH/CS-1.0 and HH/CS-1.5 exhibited broader deformation regions after reaching maximum stress, suggesting relatively better toughness and structural cohesion compared with the highly fiber-loaded samples. Overall, the HH/CS ratio governed the trade-off between structural rigidity and mechanical integrity. Among the investigated samples, HH/CS-2.0 exhibited the most balanced mechanical performance, providing sufficient stiffness while maintaining structural cohesion, which is beneficial for repeated oil sorption and regeneration.

#### 3.1.5 Oil sorption performance as a function of HH/CS ratio

**Fig. 4:**
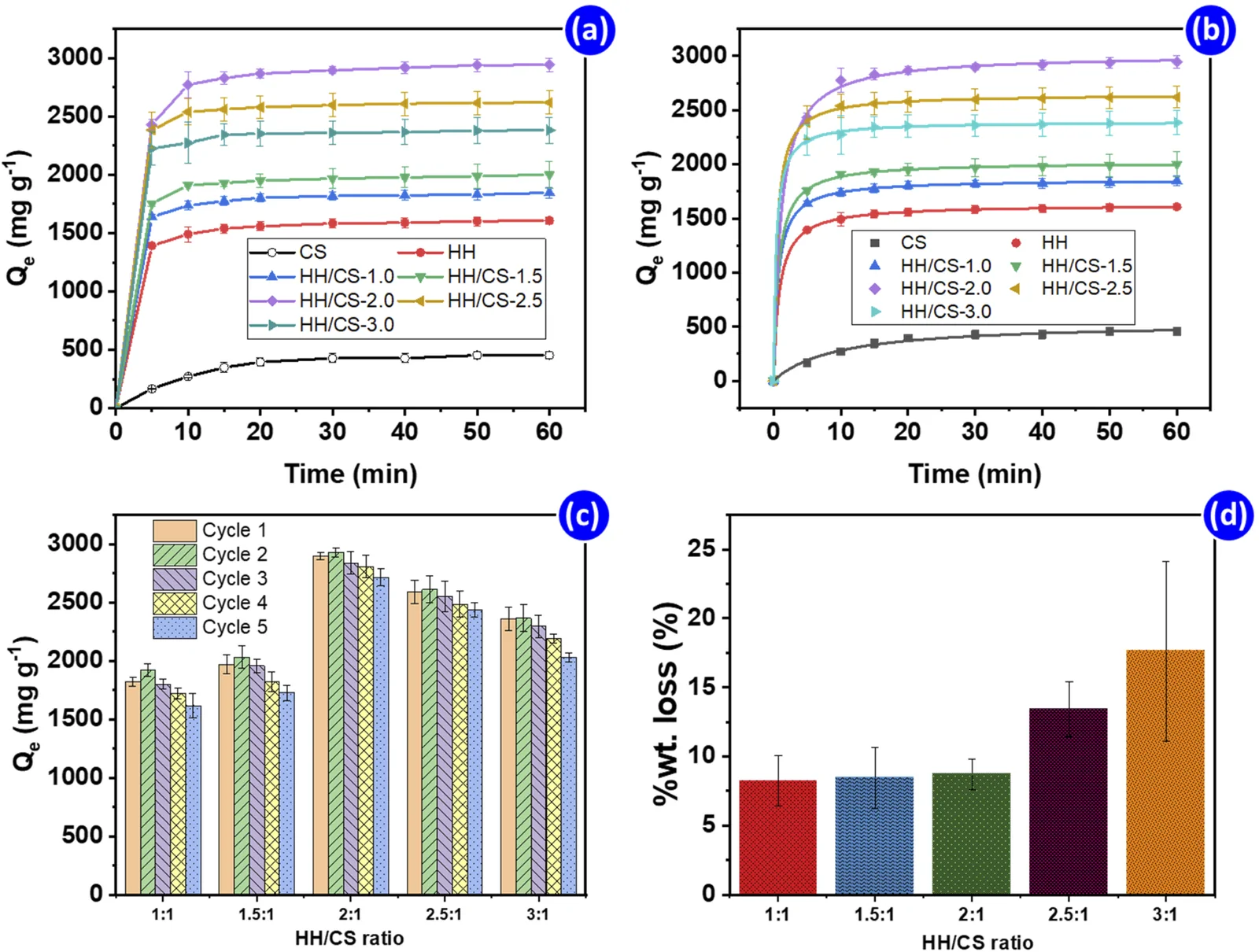
Oil sorption and reusability performances of HH/CS composite mats with different HH/CS mass ratios: (a) oil sorption capacities of the composite mats as a function of adsorption time using fresh motorcycle engine oil under ambient conditions; (b) nonlinear PSO kinetic fitting of the oil sorption data for CS, HH, and HH/CS composite mats; (c) reusability performances of the HH/CS composite mats during five consecutive adsorption–desorption cycles; (d) mass loss percentages of the composite mats after five reuse cycles.

Efficient oil sorbent materials should possess not only high sorption capacity but also rapid uptake kinetics, good structural integrity, and excellent reusability. Therefore, the oil sorption performance of the HH/CS composite mats with different HH/CS mass ratios was systematically evaluated to identify the optimal composition. As shown in Fig. 4a, all HH-containing composite mats exhibited substantially higher oil sorption capacities than the pristine CS mat, highlighting the beneficial effect of HH incorporation on oil sorption performance. The pure CS membrane displayed very limited adsorption performance, reaching only approximately 454 mg g^−1^ after 60 min, whereas raw HH fibers achieved capacities exceeding 1600 mg g^−1^. The incorporation of HH into the CS matrix significantly enhanced the adsorption performance, with all composite mats exhibiting rapid oil uptake during the initial adsorption stage (within the first 5–10 min), followed by a slower approach toward equilibrium. This behavior could be attributed to the rapid occupation of readily accessible surface pores and interconnected capillary channels during the early stage, followed by gradual diffusion of oil into the deeper internal structure of the composite network.

Among the investigated samples, HH/CS-2.0 exhibited the highest oil sorption capacity throughout the adsorption process, reaching 2896.95 mg g^−1^ after 30 min and approaching equilibrium at approximately 2942.90 mg g^−1^ after 60 min. In contrast, further increasing the HH content to HH/CS-2.5 and HH/CS-3.0 resulted in lower adsorption capacities despite the higher fiber loading. This observation suggests that oil sorption performance was governed not only by surface wettability but also by the overall structural organization and stability of the three-dimensional composite network. Although higher hydrophobicity is beneficial for selective oil sorption, the overall adsorption performance of porous fibrous sorbents also depends on oleophilic interactions with hydrocarbons, accessible pore volume, interconnected capillary pathways, fiber packing density, and structural integrity. Accordingly, the relatively low apparent WCA of HH/CS-1.0 did not compromise its oil sorption performance because the interconnected porous architecture still facilitated efficient oil transport and retention. As discussed previously, excessive HH incorporation led to thicker and more loosely interconnected structures with reduced mechanical integrity and poorer matrix continuity. Such structures may decrease the effective capillary transport and reduce oil retention stability within the porous network. Conversely, the HH/CS-2.0 sample likely provided the most balanced combination of accessible porosity, interconnected capillary channels, oil affinity, structural cohesion, and sufficient hydrophobicity, thereby maximizing oil sorption performance.

Notably, most samples achieved near-equilibrium adsorption within approximately 30 min, indicating rapid sorption kinetics of the HH/CS composite mats. Therefore, an adsorption time of 30 min was selected for subsequent adsorption and recycling experiments.

To further elucidate the sorption mechanisms, the experimental kinetic data were fitted using nonlinear pseudo-first-order (PFO) and pseudo-second-order (PSO) kinetic models. The nonlinear PSO fitting curves are presented in Fig. 4b, whereas the corresponding PFO fitting results are provided in Fig. S1 for comparison. The fitted kinetic parameters are summarized in Table S3. The PSO model consistently exhibited higher correlation coefficients (*R*^2^ = 0.99914–0.99995) than the PFO model (*R*^2^ = 0.99672–0.99933), together with lower fitting errors for most composite samples. Moreover, the equilibrium sorption capacities (*q*_e,cal_) predicted by the PSO model closely matched the experimentally observed values. Although the PSO model is conventionally associated with chemisorption-controlled processes in dilute adsorption systems, its superior fit in the present study should not be interpreted as evidence of chemisorption. Instead, it likely reflects the combined effects of capillary-driven oil uptake, viscous flow resistance within the porous structure, and the gradual saturation of the interconnected fibrous network.

The reusability performances of the composite mats were further evaluated through five consecutive adsorption–desorption cycles using *n*-hexane as the extraction solvent, and the results are presented in Fig. 4c. Overall, all HH/CS composite mats retained relatively high adsorption capacities after repeated use, indicating good solvent resistance and structural durability of the crosslinked fibrous network. Among them, HH/CS-2.0 again demonstrated the best recycling stability, maintaining an adsorption capacity of 2714.89 mg g^−1^ after five cycles, corresponding to approximately 93.7% retention of its initial adsorption capacity. In comparison, HH/CS-3.0 exhibited a more pronounced decline in adsorption performance during repeated cycling, decreasing from 2359.43 to 2028.34 mg g^−1^ after five cycles.

The superior recyclability of HH/CS-2.0 could be attributed to its optimized balance between fiber reinforcement and matrix cohesion. In contrast, the thicker HH/CS-2.5 and HH/CS-3.0 mats likely increased the internal transport distance of oil, while excessive HH loading weakened the continuity of the CS matrix and reduced interfacial adhesion between fibers and polymer chains. Together, these effects reduced oil retention and weakened the structural robustness of the composite during repeated adsorption–desorption cycles. At moderate HH loading, the interconnected HH fibers effectively reinforced the CS framework and maintained pore stability during repeated solvent extraction and drying processes. However, excessive HH incorporation promoted fiber detachment and local collapse of the porous architecture during repeated use.

This interpretation was further supported by the mass-loss analysis shown in Fig. 4d. The HH/CS-1.0, HH/CS-1.5, and HH/CS-2.0 samples exhibited relatively low mass losses after five cycles, ranging from 8.22% to 8.69%, whereas HH/CS-2.5 and HH/CS-3.0 displayed significantly higher mass losses of 13.41% and 17.63%, respectively. The increased material loss at high HH content suggests weaker structural stability and greater detachment of loosely bound fibers from the composite matrix during repeated adsorption–desorption treatments. These findings further confirm that the HH/CS-2.0 composition provided the most favorable combination of adsorption capacity, recyclability, and structural stability among the investigated samples, and therefore this sample was selected as the optimized composite mat for subsequent characterization and oil sorption studies.

### 3.2. Structural and physicochemical characterization of the optimized composite mat

#### 3.2.1 FTIR analysis

**Fig. 5:**
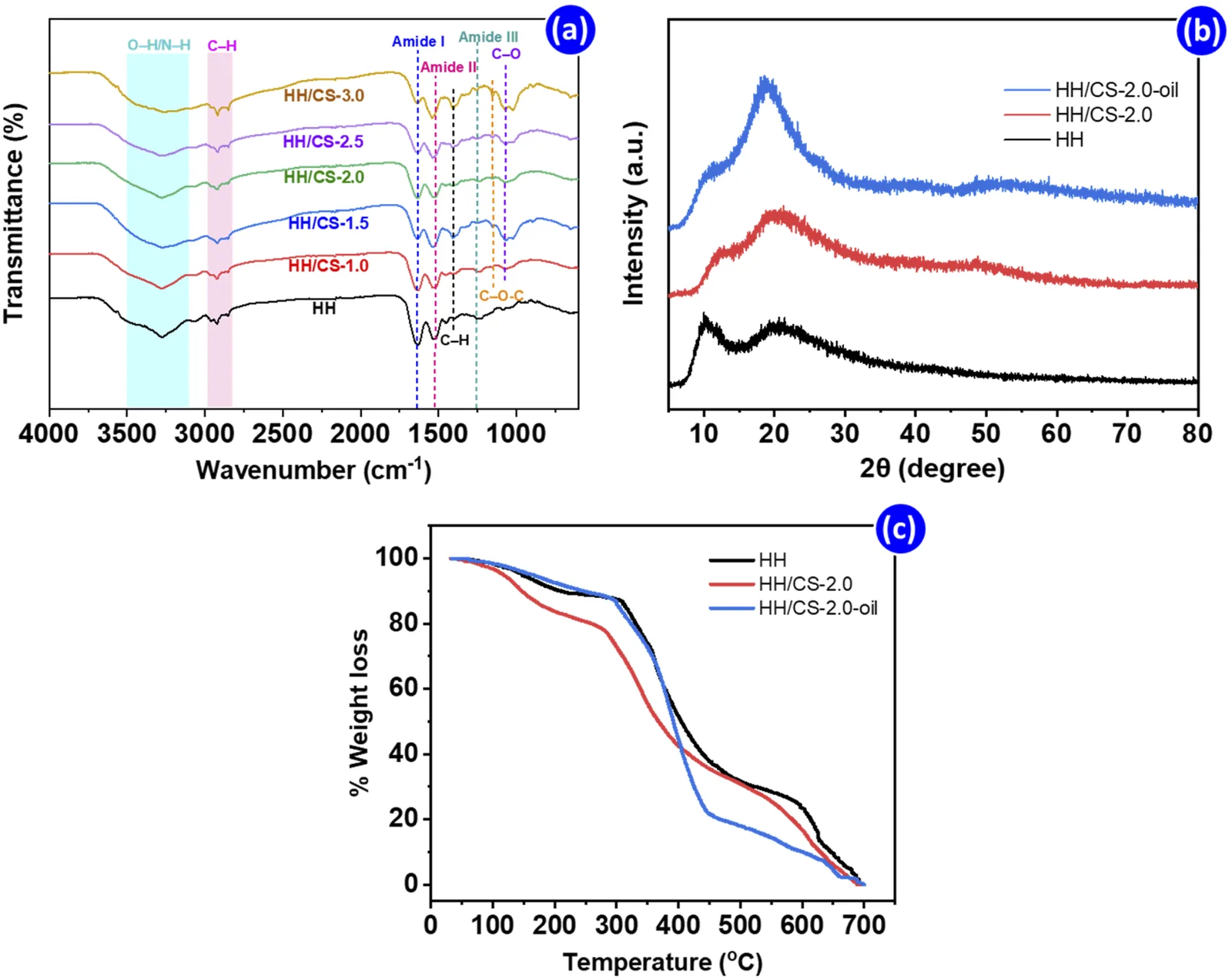
Structural and physicochemical characterization of the HH/CS composite mats: (a) FTIR spectra of pristine HH and HH/CS composite mats with different HH/CS ratios; (b) XRD patterns of pristine HH, optimized HH/CS-2.0 composite mat, and oil-loaded HH/CS-2.0 mat; and (c) TGA curves of pristine HH, HH/CS-2.0 composite mat, and oil-loaded HH/CS-2.0 sample.

FTIR spectroscopy was employed to investigate the chemical structures and intermolecular interactions within the HH/CS composite mats, and the corresponding spectra are presented in Fig. 5a. The characteristic absorption bands and their assignments are summarized in Table 1. Overall, the spectra confirmed the successful incorporation of HH fibers into the CS matrix and provided evidence of intermolecular interactions between keratin and CS components within the composite structure.

**Table 1:** Characteristic FTIR peak positions (cm^−1^) of HH and HH/CS composite samples

Assignments	HH	HH/CS-1.0	HH/CS-1.5	HH/CS-2.0	HH/CS-2.5	HH/CS-3.0	Molecular origin
O–H/N–H	3200–3500	3200–3500	3200–3500	3200–3500	3200–3500	3200–3500	Stretching of –OH and –NH groups
C–H stretching	2920, 2850	2920, 2850	2920, 2850	2920, 2850	2920, 2850	2920, 2850	Aliphatic CH_2_/CH_3_ stretching
Amide I	1630	1630	1630	1630	1630	1630	C=O stretching (peptide bond)
Amide II	1520	1530	1538	1538	1535	1538	N–H bending and C–N stretching
C–H bending	1400	1400	1407	1400	1402	1405	CH_2_/CH_3_ bending vibration
Amide III	1230	1235	1250	1239	1250	1250	Combined C–N stretching and N–H bending
C–O–C	—	1146	1151	1150	1150	1150	Asymmetric stretching of glycosidic linkage
C–O	—	1070	1065	1067	1066	1069	C–O stretching of polysaccharide backbone

The raw HH sample exhibited several characteristic absorption bands associated with keratin protein structures. The broad band observed in the range of 3200–3500 cm^−1^ was assigned to the overlapping stretching vibrations of O–H and N–H groups originating from amino acid residues and adsorbed moisture. The peaks at 2920 and 2850 cm^−1^ corresponded to the asymmetric and symmetric stretching vibrations of aliphatic C–H groups.^24^ In addition, the characteristic amide bands of keratin were clearly observed, including the amide I band at approximately 1630 cm^−1^ (C=O stretching vibration of peptide bonds), the amide II band at approximately 1520 cm^−1^ (N–H bending coupled with C–N stretching), and the amide III band around 1230 cm^−1^, which is commonly associated with combined C–N stretching and N–H bending vibrations of protein backbones.^25,26^

After incorporation of CS, several characteristic polysaccharide-related bands appeared in all composite samples, including those at approximately 1146–1151 and 1065–1070 cm^−1^, corresponding to the asymmetric stretching of glycosidic C–O–C linkages and C–O stretching vibrations of the CS backbone, respectively.^27,28^ Their absence in pristine HH and consistent presence in all composites confirmed the successful incorporation of CS into the fibrous network.

More importantly, noticeable shifts in the amide-related bands were detected after composite formation. Compared with pristine HH, the amide II band shifted from 1520 to 1530–1538 cm^−1^, while the amide III band shifted from 1230 to 1235–1250 cm^−1^. These spectral shifts indicate the presence of strong intermolecular interactions, primarily hydrogen bonding accompanied by partial electrostatic interactions, between the keratin functionalities of HH fibers and the amino/hydroxyl groups of the CS matrix, consistent with the proposed structural interactions shown in Fig. 1a. Similar amide band broadening and spectral profile changes resulting from keratin–CS complexation have also been reported by Fras Zemljič *et al.*^26^

In addition, the broad O–H/N–H stretching region became slightly wider and less symmetric with increasing HH content, particularly for HH/CS-2.5 and HH/CS-3.0. This behavior reflects the overlapping contributions of hydrophilic groups from both polymers and indicates the formation of increasingly heterogeneous hydrogen-bonding environments within the composite network due to the higher fiber loading and more complex interfacial interactions between HH fibers and CS chains. Similar spectral broadening phenomena have been reported in other keratin–polysaccharide composite systems, where extensive intermolecular hydrogen bonding contributes to structural stabilization of the hybrid network.^29,30^

Although TPP was employed as the ionic crosslinker, its characteristic phosphate vibrations were not clearly resolved because of their relatively low content and overlap with the strong amide III and polysaccharide bands in the 1000–1250 cm^−1^ region. Likewise, the weak S–S vibration of keratin was not distinctly observed owing to its inherently low spectral intensity. Nevertheless, the overall FTIR results support the successful incorporation of HH into the CS matrix and the establishment of strong intermolecular interactions responsible for the formation of the stable composite network.

#### 3.2.2 XRD analysis

XRD analysis was performed to investigate the structural organization and diffraction characteristics of the HH fibers, the optimized HH/CS-2.0 composite mat, and the oil-loaded HH/CS-2.0 sample after adsorption. As shown in Fig. 5b, the pristine HH sample exhibited two broad diffraction peaks centered at approximately 2*θ* = 10° and 20°, which are characteristic features of partially crystalline α-keratin structures commonly observed in HH fibers, corresponding to the interhelix and axial periodicities of the microfibrils, respectively.^31^ The broad nature of these peaks indicates the coexistence of ordered crystalline domains and amorphous protein regions within the keratin matrix.

After incorporation into the CS matrix and TPP crosslinking, the HH/CS-2.0 composite exhibited a noticeable broadening of the diffraction region around 20°, accompanied by a reduction in the relative intensity of the low-angle diffraction feature near 10°. This behavior suggests a partial relaxation of the native keratin packing structure and the formation of a more disordered semi-amorphous network. Such structural rearrangement may be associated with extensive intermolecular interactions among keratin chains, CS macromolecules, and ionic crosslinking sites generated by TPP, including hydrogen bonding, electrostatic attraction, and hydrophobic interactions. These interactions are known to alter polymer chain packing and promote formation of more relaxed amorphous architectures in protein–polysaccharide composite systems.^32,33^ The reduction in structural ordering observed in the present study is also consistent with the FTIR results, which revealed strong intermolecular interactions between HH and CS components.

The predominance of broad diffraction features in the HH/CS-2.0 composite further indicates that the prepared mat possessed a largely amorphous and loosely packed architecture, which is advantageous for oil sorption applications because amorphous regions generally facilitate liquid diffusion and adsorption accessibility. Similar reductions in crystallinity have been reported in other keratin–polysaccharide hybrid systems, where polymer blending and intermolecular hydrogen bonding suppressed ordered chain packing and promoted formation of flexible adsorption networks.^33^

After oil adsorption, the XRD pattern of HH/CS-2.0-oil retained the broad amorphous diffraction profile without the appearance of new crystalline peaks, indicating that the adsorption process did not destroy the structural framework of the composite mat. However, a slight increase in the intensity together with subtle broadening of the diffraction feature around 20° was observed after oil sorption. This change is consistent with the presence of absorbed oil within the porous composite network, although it should not be interpreted as evidence of a new crystalline phase or a significant structural rearrangement. This observation implies that oil uptake mainly occurred through physical absorption and capillary diffusion within the interconnected fibrous structure rather than through crystallization or irreversible structural transformation. The preserved amorphous architecture after adsorption also supports the good reusability and structural stability observed during cyclic oil sorption experiments.

#### 3.2.3 TGA analysis

The thermal stability and decomposition behavior of pristine HH, optimized HH/CS-2.0 composite mats, and oil-loaded HH/CS-2.0 mats were investigated by TGA, as shown in Fig. 5c. All samples exhibited multi-step thermal degradation behavior, reflecting the complex composition and hierarchical structure of the keratin–CS network.

The initial weight loss below approximately 120 °C was relatively small and can be attributed to the evaporation of physically adsorbed moisture and loosely bound water molecules associated with hydrophilic functional groups in keratin and CS. The HH/CS-2.0 composite exhibited slightly higher mass loss in this region compared with pristine HH, likely due to the abundant hydroxyl and amino groups introduced by the CS matrix, which enhanced moisture retention through hydrogen bonding interactions.

For pristine HH, the major decomposition stage occurred mainly within the temperature range of approximately 300–450 °C, corresponding to the extensive thermal degradation of keratin chains, including cleavage of peptide bonds, decomposition of amino acid side groups, and breakdown of the α-keratin structure.^34^ Notably, this major stage was preceded by a subtle thermal transition around 160–250 °C, which is typically associated with the cleavage of disulfide (S–S) bonds and the denaturation of the α-helix conformation. The relatively broad degradation profile is characteristic of proteinaceous biomaterials containing heterogeneous sulfur-containing amino acids and highly crosslinked structures.^35^

After incorporation into the CS matrix, the HH/CS-2.0 composite displayed a noticeably altered degradation pathway. The onset of thermal degradation shifted toward lower temperatures, and a more pronounced weight-loss region was observed between approximately 120 and 280 °C. This behavior suggests the early thermal decomposition of the CS component within the interconnected hybrid network, as well as the evaporation of a larger amount of bound water trapped inside the amorphous composite structure, which strongly correlates with the XRD analysis.

Interestingly, the oil-loaded HH/CS-2.0 sample exhibited a highly distinct thermal degradation profile compared with both the pristine HH and the fresh composite mat. In the lower temperature range (below 300 °C), the oil-loaded mat showed a delayed weight loss relative to the fresh HH/CS-2.0 mat. This can be attributed to the hydrophobic oil layer coating the matrix surface, which restricted the diffusion and evaporation of internal moisture. However, a remarkably rapid and sharp weight loss was observed within the range of approximately 300–450 °C, where the curve dropped precipitously below those of the other samples, leaving a much lower residual mass near 450 °C. This phenomenon is a direct consequence of the thermal volatilization and cracking of the absorbed hydrocarbon chains from the motor oil. The simultaneous decomposition of the oil molecules and the polymer backbone accelerated the overall mass reduction process. This distinct thermal response is consistent with the presence of absorbed hydrocarbons within the porous HH/CS composite and supports the successful retention of oil after sorption.

#### 3.2.4 Morphological and elemental analysis

The surface morphology and microstructural characteristics of the optimized HH/CS-2.0 composite mat were investigated by SEM analysis, while elemental composition and spatial distribution were evaluated using EDX spectroscopy and elemental mapping. In addition, the morphology and elemental characteristics of pristine HH fibers are provided in Fig. S2 for comparison.

For the pristine HH fibers, the SEM micrograph and the corresponding elemental mapping (Fig. S2, SI) clearly display the characteristic cylindrical, fibrous morphology of HH with distinct overlapping cuticle scales on the outermost surface.^36^ The EDX spectrum confirms the proteinaceous nature of the fibers, with carbon, oxygen, nitrogen, and sulfur identified as the major constituent elements. Quantitative analysis showed that carbon and oxygen were dominant, accounting for 53.92 wt% and 26.55 wt%, respectively. Notably, sulfur was uniformly distributed throughout the fiber structure, which can be attributed to sulfur-containing amino acids such as cystine and cysteine present in keratin proteins. The presence of sulfur is considered a characteristic chemical signature of keratinous materials due to the abundance of disulfide (S–S) linkages within the hair structure.

**Fig. 6:**
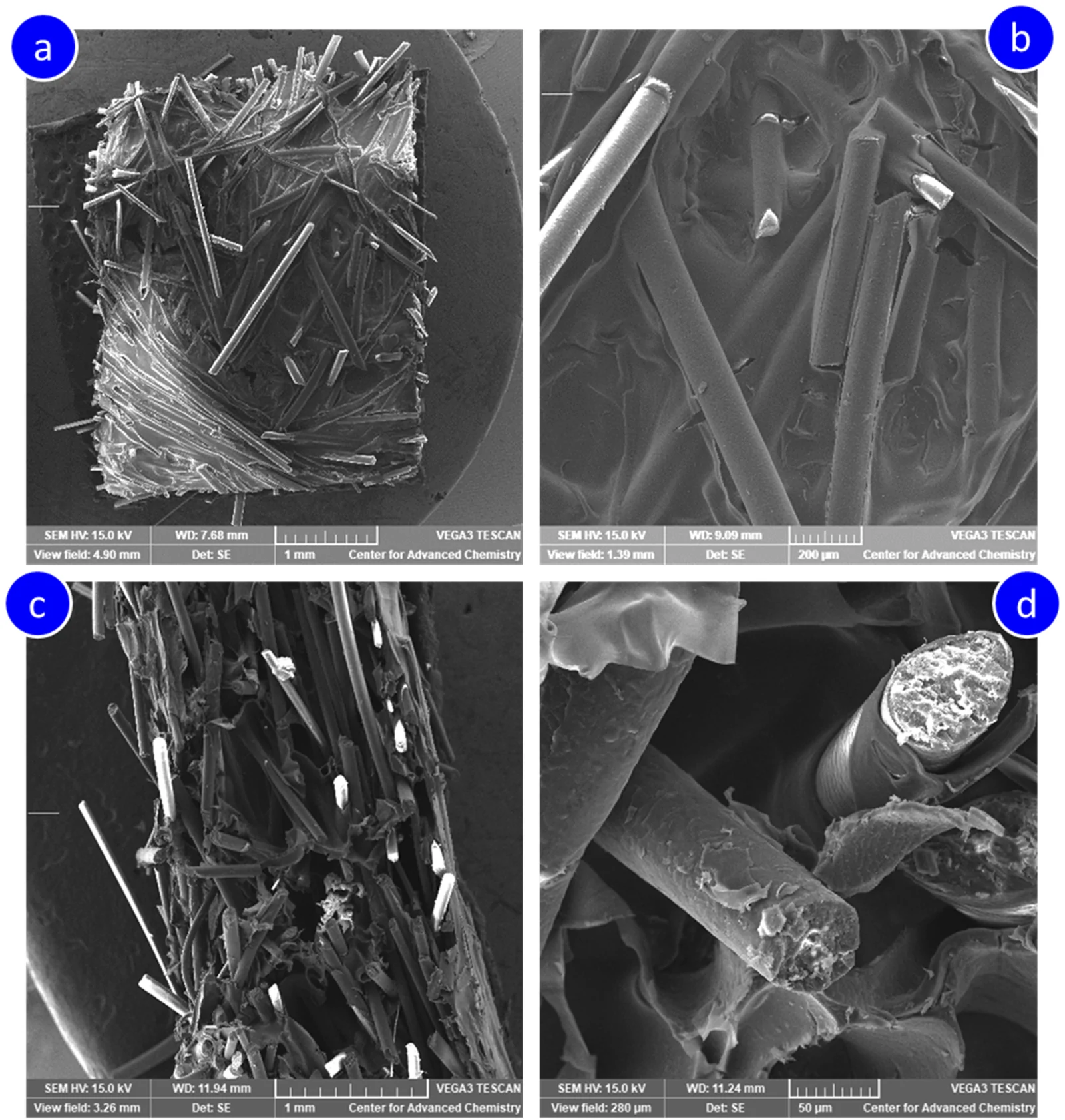
SEM micrographs of the optimized HH/CS-2.0 composite mat: (a) low-magnification surface view, (b) high-magnification surface view showing the embedding of HH fibers within the CS matrix, (c) cross-sectional view of the mat, and (d) high-magnification cross-sectional view illustrating the internal fiber arrangement and matrix–fiber adhesion.

After incorporation into the CS matrix and subsequent TPP crosslinking, significant microstructural evolution was observed in the optimized HH/CS-2.0 composite mat (Fig. 6). The low-magnification SEM image (Fig. 6a) demonstrates the formation of a highly porous three-dimensional network composed of randomly distributed and interconnected HH segments embedded within the CS matrix. At higher magnification (Fig. 6b), the CS phase can be clearly observed coating and bridging adjacent HH fibers, thereby forming an interconnected hybrid architecture rather than a compact dense film. This configuration generated a rough hierarchical surface morphology containing abundant inter-fiber voids and capillary channels, which are advantageous for rapid oil penetration and retention during sorption processes.

The cross-sectional morphology further confirmed the formation of an interconnected porous framework (Fig. 6c and d). The embedded HH fibers remained well integrated within the continuous CS matrix without obvious fiber pull-out or severe interfacial separation. In addition, the CS coating appeared to partially surround and anchor the HH fibers, which could contribute to enhanced structural stability and reduced fiber leakage during repeated adsorption–desorption cycles. The porous internal structure observed in the cross-section is also consistent with the previously discussed reduction in apparent density and increased oil sorption capacity of the composite mats.

**Fig. 7:**
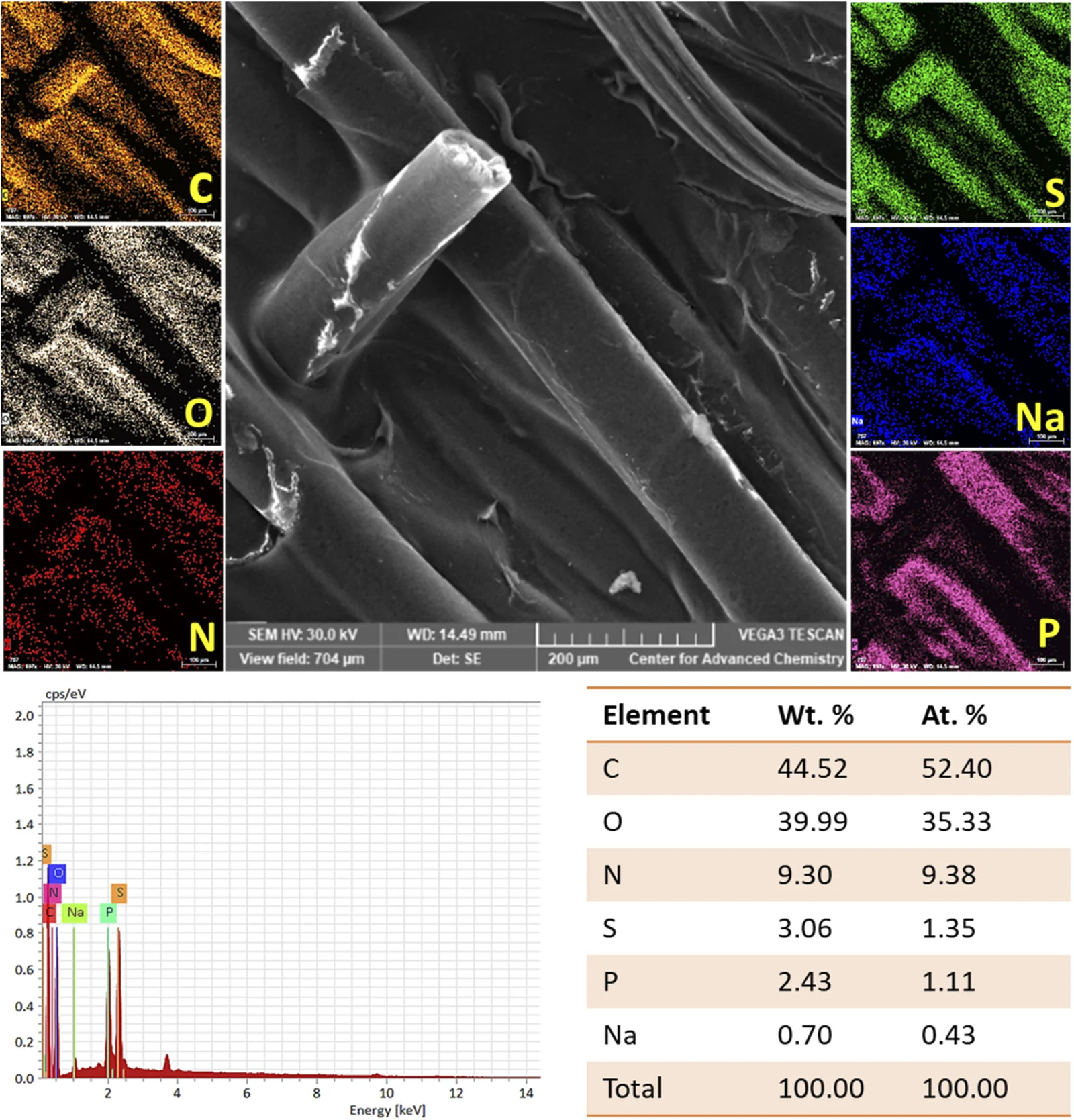
SEM micrograph, elemental mapping images (C, S, O, Na, N, and P), and EDS spectrum with corresponding elemental composition of the HH/CS-2.0 composite mat.

To further verify the successful incorporation of the TPP crosslinker and elemental distribution within the hybrid network, EDX mapping analysis was performed on the HH/CS-2.0 composite mat (Fig. 7). The mapping images revealed relatively homogeneous distributions of carbon (C), oxygen (O), nitrogen (N), sulfur (S), sodium (Na), and phosphorus (P) throughout both the fibrous regions and surrounding matrix. Compared with pristine HH fibers, the composite mat exhibited the distinct appearance of phosphorus and sodium signals, confirming the presence of sodium tripolyphosphate within the crosslinked structure. The phosphorus-containing species are associated with ionic interactions between TPP phosphate groups and protonated amino groups (–NH_3_^+^) of CS chains, thereby supporting successful ionic crosslinking within the hybrid matrix.^37^

The EDX quantitative results also demonstrated noticeable changes in elemental composition after composite formation. The carbon and nitrogen contents decreased to 44.52 wt% and 9.30 wt%, respectively, due to incorporation of the oxygen- and phosphorus-containing CS/TPP network. Importantly, nitrogen signals in the composite originated from both keratin proteins and amino groups present in CS chains, while sulfur remained primarily associated with the keratinaceous HH component. Thus, the SEM–EDX observations confirmed that the fabricated HH/CS-2.0 mat possessed a stable porous interconnected architecture with good fiber–matrix integration and homogeneous elemental distribution, which collectively provide favorable structural characteristics for efficient oil sorption and repeated reuse applications.

### 3.3. Effect of operational parameters on oil sorption performance

To further evaluate the practical applicability of the optimized HH/CS-2.0 composite mat, the effects of several operational parameters on oil sorption behavior were systematically investigated using fresh motorcycle oil as the model sorbate. Since the adsorption equilibrium time had already been established in the previous section, subsequent experiments were conducted using a contact time of 30 min. The influence of temperature, initial oil volume, mat size and mat thickness on the sorption capacity was first examined to better understand the adsorption behavior of the porous HH/CS composite system under different operating conditions.

#### 3.3.1 Effect of temperature

**Fig. 8:**
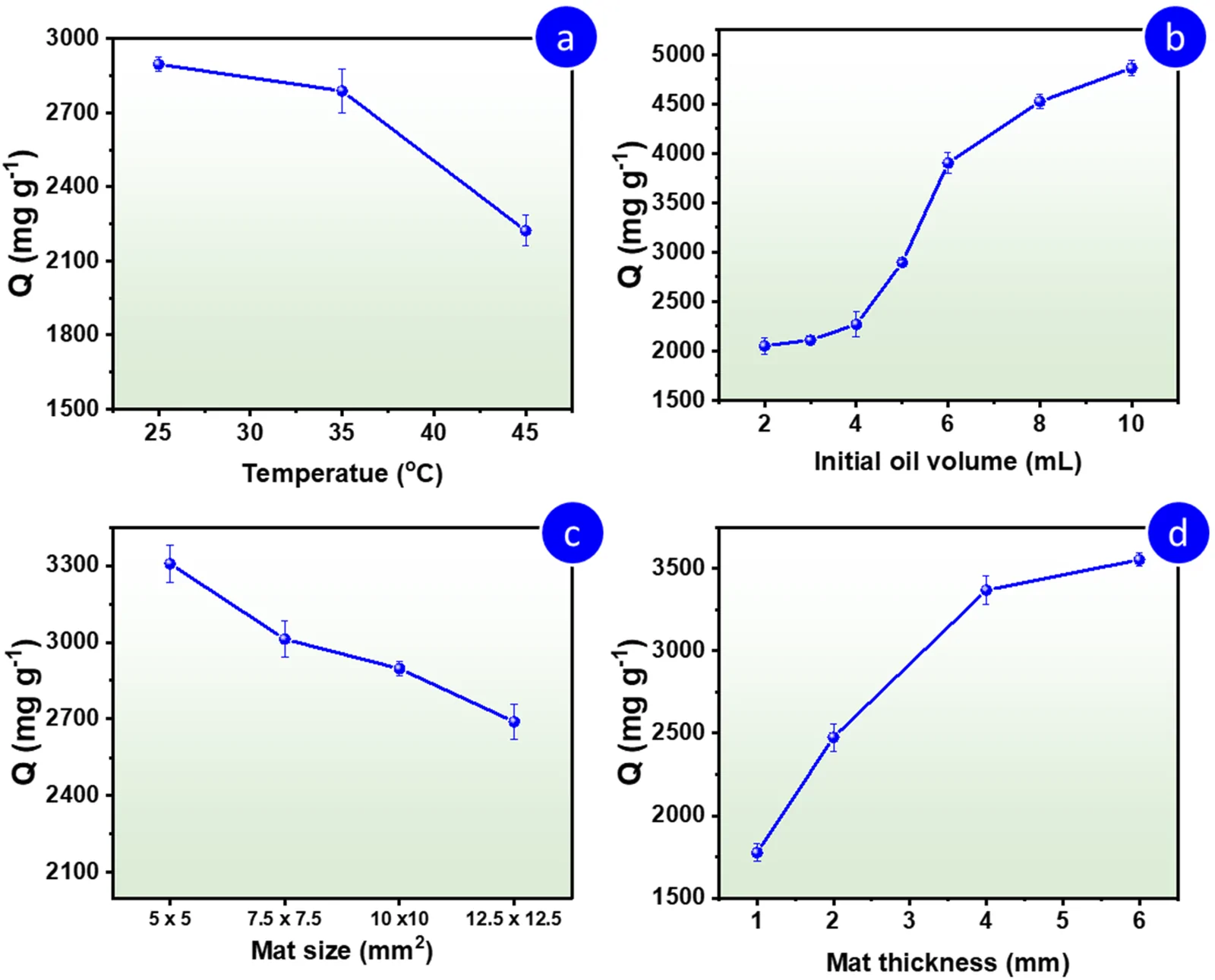
Effect of operational parameters on the oil sorption performance of the optimized HH/CS-2.0 composite mat: effect of adsorption temperature (a), initial oil volume (b), mat size (c) and mat thickness (d) on adsorption capacity.

The effect of temperature on the oil sorption performance of the HH/CS-2.0 composite mat is presented in Fig. 8a. The adsorption capacity gradually decreased from 2896.94 mg g^−1^ at 25 °C to 2788.85 mg g^−1^ at 35 °C and further declined to 2222.45 mg g^−1^ at 45 °C. This behavior suggests that elevated temperature negatively affected the oil uptake ability of the composite mat.

The reduction in sorption capacity at higher temperatures can primarily be attributed to the decrease in oil viscosity.^38^ At lower temperatures, the relatively higher viscosity of the oil facilitates retention within the porous network and capillary channels of the composite mat. However, increasing temperature lowers oil viscosity and enhances molecular mobility, thereby weakening capillary retention forces and promoting faster desorption or drainage from the interconnected porous structure. In addition, elevated temperature may partially disrupt weak physical interactions between the oil molecules and the hydrophobic domains of the composite network, resulting in lower overall oil confinement efficiency.^39^ These observations suggest that the equilibrium oil sorption capacity of the HH/CS composite is governed primarily by oil retention within the interconnected porous network rather than by the initial oil infiltration process. Although lower oil viscosity at elevated temperatures may facilitate faster penetration into the pores, the reduced viscous resistance also promotes easier drainage from the capillary channels, resulting in a lower equilibrium oil uptake.

A similar temperature-dependent decline in oil sorption performance has been reported for various porous bio-based sorbents, where capillary-driven retention and viscous trapping mechanisms play dominant roles during adsorption.^7,39,40^ The present results therefore demonstrate that effective oil sorption by the HH/CS composite depends not only on the availability of accessible pore volume but also on the ability of the interconnected fibrous network to retain viscous hydrocarbons under equilibrium conditions. Such behavior is particularly advantageous for practical oil spill remediation, which is generally performed under ambient environmental conditions.

#### 3.3.2 Effect of initial oil volume

The effect of initial oil volume on the adsorption capacity of the HH/CS-2.0 composite mat is shown in Fig. 8b. As the oil volume increased from 2 to 10 mL, the adsorption capacity continuously increased from 2050.21 mg g^−1^ to 4864.46 mg g^−1^. A particularly sharp increase was observed when the oil volume exceeded 5 mL, indicating that the accessible pore volume and interconnected capillary network of the composite mat remained far from saturation under lower oil loading conditions.

At relatively low oil volumes, the available pore structure of the composite mat were not fully utilized, resulting in comparatively lower adsorption capacity values. As the oil volume increased, more oil molecules became available to penetrate into the interconnected porous network through capillary action and surface wetting processes, thereby improving pore filling efficiency and overall oil retention.^41^ The highly porous architecture and rough fibrous morphology of the HH/CS-2.0 mat, as previously observed in the SEM analysis, likely facilitated rapid oil diffusion and effective confinement within the three-dimensional structure. These results suggest that oil uptake is governed not only by the affinity between the oil and the sorbent surface but also by the availability of interconnected pore space capable of storing the infiltrated oil. As the oil loading increased, the sorption process progressively shifted from the occupation of readily accessible surface pores to more extensive filling of the interconnected three-dimensional porous network, thereby enabling more efficient utilization of the intrinsic storage volume of the composite. Therefore, the overall sorption capacity depends on both capillary infiltration and the effective utilization of the internal porous architecture.

Furthermore, the absence of an adsorption plateau within the investigated range suggests that the intrinsic storage capacity of the HH/CS composite had not yet been fully utilized under the tested conditions. Therefore, the measured sorption capacity was primarily limited by the amount of oil available rather than by saturation of the composite structure. This behavior highlights the excellent uptake capability and storage capacity of the HH/CS hybrid structure for hydrophobic organic liquids. This result demonstrates that the composite mat can effectively accommodate increasing amounts of oil while maintaining efficient utilization of its interconnected porous framework, further supporting its applicability as a lightweight and high-capacity oil sorbent.

#### 3.3.3 Effect of mat size

The effect of composite mat size on oil sorption performance is presented in Fig. 8c. When the mat dimensions increased from 5 × 5 mm to 12.5 × 12.5 mm, the adsorption capacity expressed in normalized form (*Q*, mg g^−1^) gradually decreased from 3307.30 mg g^−1^ to 2689.35 mg g^−1^. However, the total amount of oil absorbed (*q*, mg) simultaneously increased from 96 mg to 390.77 mg with increasing mat size. This seemingly opposite trend originates from the difference between gravimetric adsorption capacity and absolute oil uptake. Larger mats possess greater overall mass and pore volume, enabling them to accommodate larger absolute amounts of oil. However, the available oil was distributed over a substantially larger sorbent volume, resulting in less efficient utilization of the internal porous structure on a mass basis. Consequently, the total absorbed oil quantity increased continuously with mat size. Nevertheless, since the adsorption capacity was normalized to the adsorbent mass (mg g^−1^), the calculated *Q* values decreased as the composite mass increased more rapidly than the increment in absorbed oil. A similar phenomenon has frequently been reported in porous sorbent systems, where increasing the sorbent mass enhances total absolute uptake but reduces the mass-normalized capacity due to the incomplete utilization of the entire porous network.^42^ From a structural perspective, increasing the lateral dimensions also increases the transport distance from the outer boundaries toward the interior of the composite mat, which may limit the accessibility of the innermost pore regions when the available oil volume is insufficient to fully occupy the interconnected porous network. These results indicate that the gravimetric sorption capacity is governed not only by the intrinsic storage capability of the composite but also by the utilization efficiency of the accessible pore volume under a given oil loading condition.

Despite the lower gravimetric sorption capacity, the larger composite mats exhibited superior absolute oil uptake because of their larger pore volume and storage capacity. These findings further demonstrate that the oil sorption performance of the HH/CS composite depends not only on the intrinsic porous architecture but also on the matching relationship between sorbent dimensions and the available oil volume.

#### 3.3.4 Effect of mat thickness

The influence of mat thickness on oil sorption performance is shown in Fig. 8d. Increasing the thickness of the HH/CS-2.0 composite mat from 1 to 6 mm significantly enhanced the adsorption capacity from 1775.99 mg g^−1^ to 3552.89 mg g^−1^. The most pronounced increase was observed when the thickness increased from 1 to 4 mm, whereas a relatively smaller improvement occurred between 4 and 6 mm.

The enhanced sorption performance with increasing thickness can be attributed to the formation of a more developed three-dimensional porous network containing additional capillary channels and internal void spaces for oil storage. Thicker mats possess larger internal volumes and higher pore accessibility, allowing greater oil diffusion and confinement within the interconnected fibrous structure. Furthermore, increasing thickness also increases the tortuous pathways inside the composite, which may help retain absorbed oil more effectively and reduce oil leakage during handling.

However, the relatively moderate increase observed beyond 4 mm suggests that the adsorption system gradually approached an oil-limited condition under the fixed oil volume employed in this study. At excessive thickness, some internal adsorption regions may not be fully utilized because the available oil becomes insufficient to completely occupy all accessible pores and capillary domains. In addition, thicker structures may partially hinder oil diffusion into deeper internal regions due to longer transport pathways. Overall, these results demonstrate that the oil sorption performance of the HH/CS composite is governed by a balance between available storage volume and effective pore utilization. While increasing thickness provides additional interconnected pore space for oil storage, the corresponding increase in transport distance gradually limits the utilization of the deepest regions under a fixed oil loading condition.

To comprehensively contextualize the performance and engineering readiness of the developed material, a multi-attribute benchmarking was conducted against recently reported bio-based sorbents, synthetic polymeric frameworks, and commercial benchmarks (Table 2). Compared with agricultural biomass sorbents such as barley straw and oil palm leaves, the HH/CS mat achieved substantially higher sorption capacity. Its performance was also comparable to or exceeded several engineered porous materials, including hydrophobic silica aerogels, Solvay-derived adsorbents, PVDF–MWCNT foams, hair-containing felt mats,^12^ and CS-modified synthetic hair composites.^10^ Although certain highly engineered materials, such as rice husk ash derivatives, cellulose/CS sponges, and commercial polypropylene sorbents, exhibited higher maximum sorption capacities under specific testing conditions, these systems generally involve more energy-intensive fabrication processes, synthetic polymer feedstocks, or limited biodegradability. In contrast, the present HH/CS composite was prepared through a facile ambient-temperature ionic crosslinking route using abundant waste HH and biodegradable CS, while simultaneously providing excellent recyclability (>90% capacity retention after five cycles) and good structural stability. These characteristics collectively highlight the potential of the developed material as a sustainable, eco-friendly, and highly promising alternative for oil spill remediation applications.

**Table 2:** Comparison of the developed HH/CS composite mat with representative bio-based and commercial oil sorbents reported in the literature

Sorbent material	Feedstock	Fabrication strategy	Target oil	Sorption capacity (mg g^−1^)	Reusability	Advantages/limitations	Ref.
Surfactant-modified barley straw	Agricultural waste	Surfactant modification (CPC)	Canola oil	576	Not reported	Simple agricultural biomass but relatively low oil uptake	43
Lauric acid-modified oil palm leaves	Agricultural waste	Fatty acid surface modification	Crude oil	1200	Not reported	Renewable biomass requiring hydrophobic modification	44
Chitosan-modified synthetic hairs	Waste synthetic hair	Chitosan precipitation onto pulverized synthetic hair particles	Crude oil	2210	3 cycles (≈59% retention)	Hair-derived composite with moderate sorption capacity and recyclability	10
Human hair/sheep wool felt mat	Waste human hair + sheep wool	Traditional felting process	Marine crude oil	3440–8330	Not systematically evaluated	Simple fabrication but performance strongly dependent on hair/wool composition	12
Hydrophobic silica aerogel	Silica	Hydrophobic surface treatment	Emulsified oil	2800	Not reported	High-performance porous material but relatively costly synthesis	45
Solvay-derived adsorbent	Industrial waste	Electrochemical recovery	Crude oil	2800	2 cycles (75% retention)	Waste-derived inorganic sorbent with limited regeneration	46
PVDF-MWCNT foam	Synthetic polymer	Salt-template foaming	Rapeseed oil	3000	10 cycles (≈300–420% capacity retained depending on regeneration method)	Excellent durability and chemical resistance but petroleum-derived and thermally processed	47
Rice husk ash	Agricultural waste	Pyrolysis	Crude oil	6200	Not reported	High adsorption capacity with simple biomass precursor	48
Cellulose/CS composite sponge	Biomass	Freeze-drying	Crude oil	5240	6 cycles	High sorption performance but requires freeze-drying fabrication	16
Commercial non-woven polypropylene sorbent	Polypropylene	Industrial non-woven processing	Lubricating oil	18 700	Reusable after centrifugation	Commercial benchmark with excellent oil uptake but non-biodegradable and fossil-base	49
HH/CS composite mat	Waste human hair + chitosan	Ambient TPP-mediated ionic crosslinking (no freeze-drying, no hydrophobic coating)	Motorcycle engine oil	4864	5 cycles (>90% retention; mass loss <9%)	Waste-derived, biodegradable composite with simple fabrication, high recyclability and structural stability	This study

### 3.4. Oil sorption behavior at the oil–water interface

**Fig. 9:**
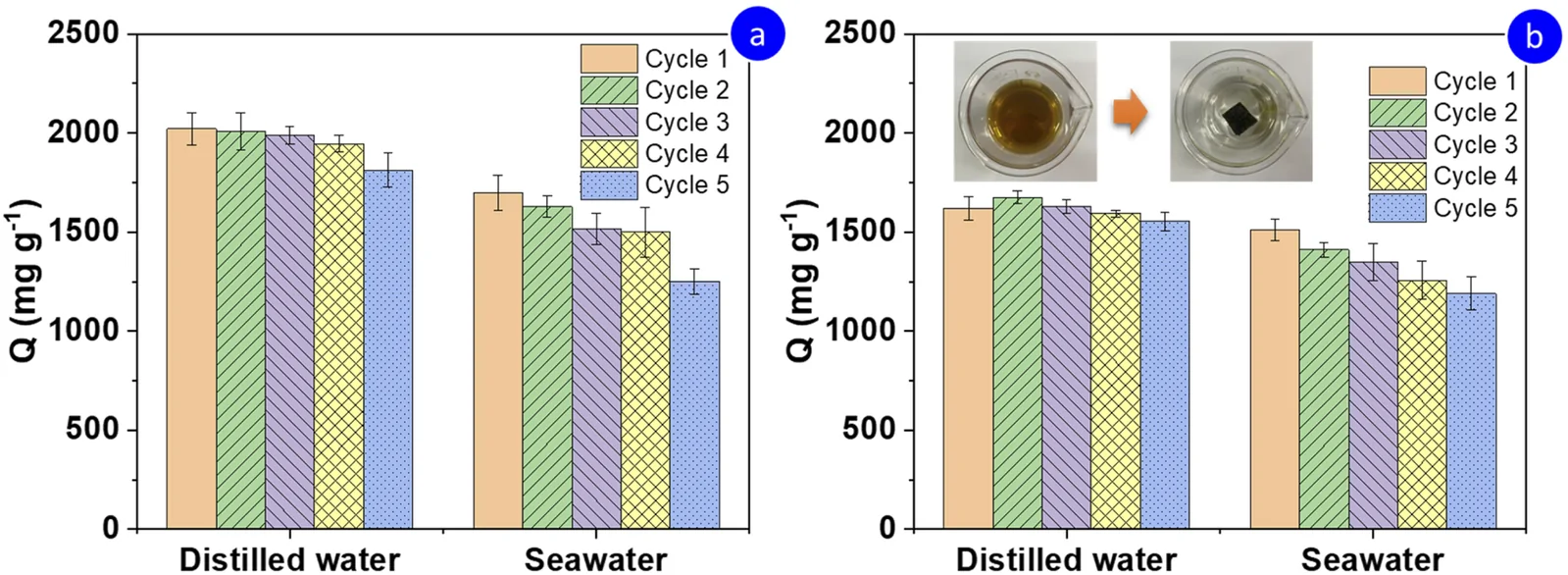
Oil sorption behavior of the optimized HH/CS-2.0 composite mat at the oil–water interface during five consecutive reuse cycles: (a) fresh motorcycle engine oil and (b) used motorcycle engine oil in distilled water and seawater systems. Insets show representative photographs of oil removal from the water surface using the composite mat.

To further evaluate the potential applicability of the developed sorbent under realistic oil spill conditions, the oil recovery performance of the optimized HH/CS-2.0 composite mat was investigated at the oil–water interface using both fresh and used motorcycle engine oils. Unlike the bulk oil sorption experiments presented in Section 3.3, these tests were performed with a thin floating oil layer on water to simulate practical oil spill remediation. Consequently, the obtained sorption capacities represent the oil recovery performance under oil–water interfacial conditions rather than the intrinsic maximum bulk oil sorption capacity of the composite. The influence of water salinity and oil properties on sorption efficiency and structural durability was systematically examined, and the results are presented in Fig. 9.

As shown in Fig. 9a, the HH/CS-2.0 mat exhibited excellent oil sorption capability toward fresh engine oil floating on water surfaces. In distilled water, the initial sorption capacity reached approximately 2021.43 mg g^−1^, followed by a gradual decrease to 1813.57 mg g^−1^ after five reuse cycles. The lower sorption capacity compared with the maximum bulk oil sorption capacity reported in Section 3.3 primarily reflects the limited availability of the floating oil layer rather than the intrinsic storage capability of the composite. Whereas the bulk oil experiments allowed continuous pore filling under excess oil, the oil–water configuration restricted capillary infiltration by limiting the amount of oil in contact with the porous matrix. Consequently, the present values provide a more realistic assessment of the oil recovery performance of the HH/CS composite under simulated oil spill conditions. A similar decreasing trend was observed in seawater, where the sorption capacity declined from 1698.09 to 1251.83 mg g^−1^. Despite the reduction after repeated use, the composite mat still maintained relatively high oil uptake ability, indicating good structural integrity and interfacial stability during cyclic operation.

The lower sorption capacities observed in seawater compared with distilled water are consistent with the well-established sorption behavior of hydrophobic organic compounds in saline media. The presence of dissolved salts may modify interfacial properties and partially hinder capillary-driven oil penetration into porous sorbents, thereby reducing oil transport and retention within the composite network.^50,51^

For used engine oil (Fig. 9b), the adsorption capacities were generally lower than those obtained for fresh oil under identical conditions. In distilled water, the sorption capacity ranged from 1620.10 to 1553.15 mg g^−1^ over five cycles, whereas in seawater the values decreased from 1511.36 to 1189.32 mg g^−1^. This behavior was likely associated with the more complex chemical composition of used engine oil, which contains oxidized hydrocarbons, carbonaceous residues, metal particles, and degradation by-products generated during engine operation.^52^ These contaminants could hinder oil diffusion into the interconnected porous structure, partially block sorption pathways, and alter interfacial wetting behavior within the composite network.

Interestingly, although used oil exhibited lower adsorption capacity, the decline during repeated reuse cycles was less pronounced in distilled water compared with fresh oil. This observation may be attributed to the stronger retention of viscous heavy hydrocarbon fractions within the porous structure, which reduced desorption efficiency while stabilizing residual oil entrapment during subsequent cycles.

The structural durability of the composite mat after repeated oil–water sorption cycles was further evaluated through mass loss analysis. The HH/CS-2.0 mat exhibited relatively low weight loss values after five reuse cycles, with reductions of 7.70 ± 2.92% and 8.47 ± 0.66% for fresh oil sorption in distilled water and seawater, respectively. Similarly, for used oil sorption, the corresponding weight losses were 8.59 ± 2.93% and 5.84 ± 0.82%. These relatively small mass reductions indicate that the hybrid keratin–CS framework possessed good mechanical stability and resistance against structural disintegration under repeated immersion, solvent extraction, drying, and regeneration processes. The HH/CS-2.0 composite mat maintained high oil recovery efficiency and good structural stability under both distilled water and seawater conditions, demonstrating its potential for repeated oil spill recovery in different aquatic environments.

### 3.5. Oil sorption mechanism

The oil sorption behavior of porous bio-based sorbents is generally governed by multiple synergistic factors, including surface roughness, interconnected pore channels, capillary-driven transport, hydrophobic affinity, and internal diffusion pathways within the three-dimensional network structure. In hybrid fibrous systems, oil uptake typically occurs through rapid penetration of oil into interconnected voids, followed by physical retention within the porous architecture and interfacial interactions between hydrocarbon molecules and the sorbent surface.^53,54^ Therefore, elucidating the sorption mechanism of the HH/CS composite mats requires comprehensive spectroscopic and compositional analyses before and after oil uptake. In this study, FTIR, XPS, and GC-MS analyses were employed to evaluate the evolution of surface functional groups, elemental composition, and hydrocarbon retention, thereby providing insight into the physicochemical processes governing oil sorption.

**Fig. 10:**
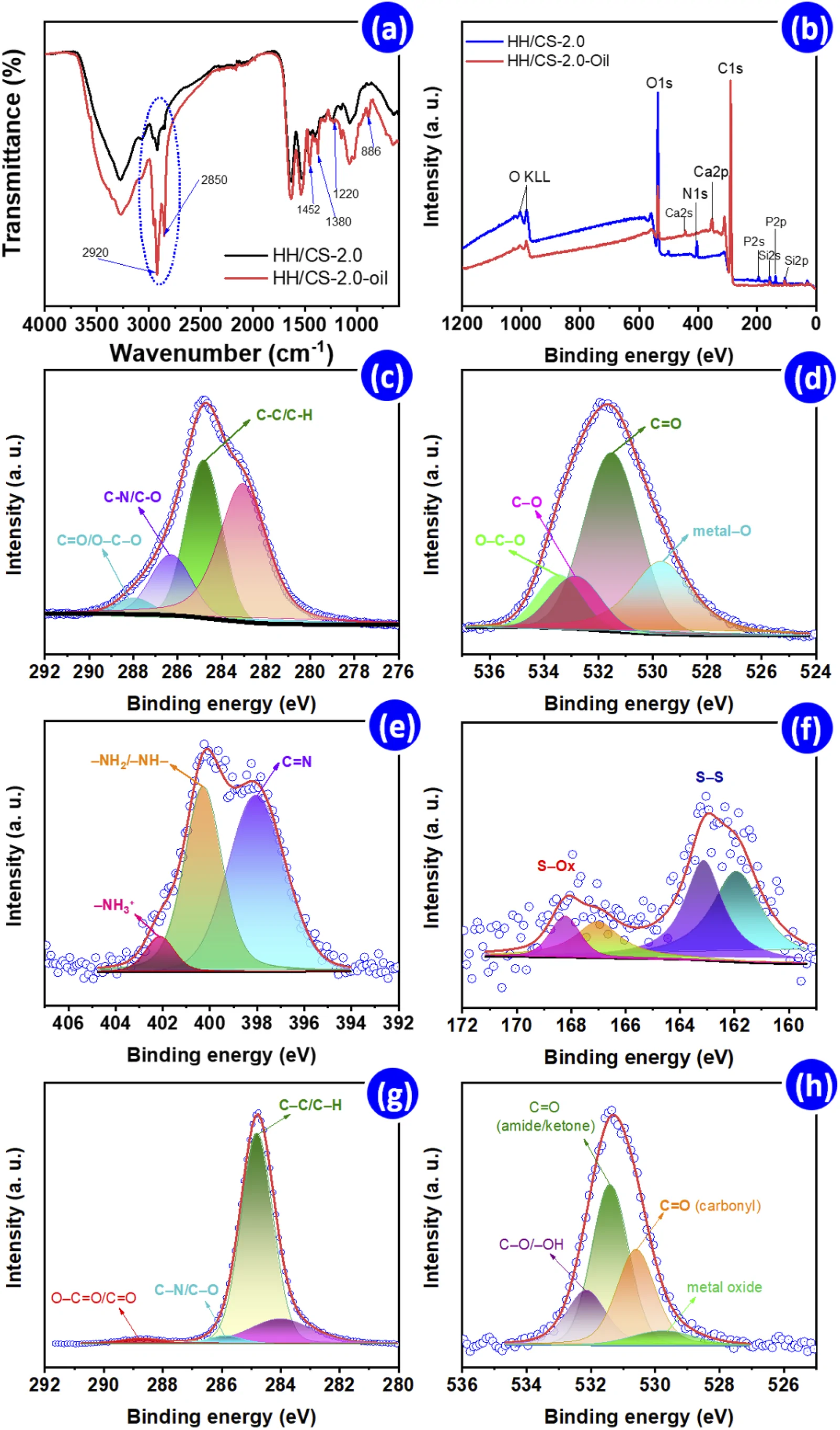
FTIR and XPS analyses of the HH/CS-2.0 composite mats before and after oil sorption: (a) FTIR spectra, (b) XPS survey spectra, deconvoluted high-resolution XPS spectra of pristine HH/CS-2.0 for (c) C 1s, (d) O 1s, (e) N 1s, and (f) S 2p, and deconvoluted high-resolution spectra of oil-loaded HH/CS-2.0 for (g) C 1s and (h) O 1s.

The FTIR spectra of the HH/CS-2.0 mat before and after oil sorption are presented in Fig. 10a. Following the adsorption process, several significant spectral changes were observed, indicating the successful uptake and confinement of oil molecules within the porous composite matrix. Most notably, the absorption bands located at approximately 2920 and 2850 cm^−1^ became remarkably sharper and more intense after adsorption. These bands are attributed to the asymmetric and symmetric stretching vibrations of aliphatic CH_2_ and CH_3_ groups, respectively, which are characteristic spectral signatures of the long-chain hydrocarbon compounds commonly present in lubricating oils. In addition, the enhanced band observed near 1452 cm^−1^ corresponds to the bending vibration of aliphatic C–H groups, while the newly emerged peak at 886 cm^−1^ is assigned to the aliphatic C–H out-of-plane bending vibrations, further supporting the substantial accumulation and dense confinement of hydrocarbon species within the HH/CS framework.^55^ The dramatic increase in the relative intensity of these aliphatic bands indicates that substantial amounts of oil were successfully retained inside the interconnected porous architecture of the composite mat. Meanwhile, the characteristic amide-related absorption bands associated with keratin and CS remained detectable after sorption, suggesting that the fundamental chemical backbone of the HH/CS hybrid structure was well-preserved during the adsorption process. Slight variations in local peak profiles within the fingerprint region (1500–1000 cm^−1^) were also observed, which may be related to weak intermolecular interactions between the adsorbed hydrocarbon molecules and the matrix's functional groups. Overall, the coexistence of the preserved polymeric framework and the intensified hydrocarbon-associated vibrations demonstrates that oil sorption predominantly occurred through physical entrapment, capillary infiltration, and hydrophobic interactions within the hierarchical porous network, rather than through irreversible chemical transformation of the sorbent structure. These observations further suggest that the amino, hydroxyl, amide, and disulfide functionalities of the HH/CS framework primarily contribute to the structural stability of the composite network, whereas oil retention is governed predominantly by capillary confinement together with weak hydrophobic and van der Waals interactions rather than the formation of new covalent bonds.

To evaluate the evolution of the surface elemental composition and adsorption mechanism of the hybrid mat before and after oil sorption, XPS survey and high-resolution spectra were systematically analyzed. The fully annotated survey spectra are presented in Fig. S3, while the simplified survey spectra and corresponding elemental compositions are shown in Fig. 10b and Table 3, respectively. The survey spectrum of pristine HH/CS-2.0 exhibited characteristic signals of the biopolymer-based composite, dominated by C 1s (62.76 at%), O 1s (24.77 at%), and N 1s (5.15 at%). In addition, distinct P 2p (2.86 at%) and Na 1s (0.90 at%) signals were detected, confirming the successful incorporation of the TPP ionic crosslinker within the hybrid matrix, in good agreement with the SEM–EDX results. A weak S 2p signal (0.36 at%) associated with sulfur-containing keratin residues from the HH fibers was also observed. The survey spectrum also exhibited a minor Si 2p feature, which is reasonably attributed to trace silica-containing contaminants remaining on the surface of waste human hair after collection and cleaning, rather than to any constituent of the HH/CS composite.

**Table 3:** Surface elemental composition (atomic%) of HH/CS-2.0 before and after oil adsorption

Element	Before (%)	After (%)
C 1s	62.76	86.58
N 1s	5.15	0.59
O 1s	24.77	9.85
Na 1s	0.90	—
Si 2p	2.41	1.40
P 2p	2.86	0.25
S 2p	0.36	0.10
Ca 2p	0.28	1.07
Zn 2p_3/2_	—	0.13
Mo 3d	0.02	0.04

After oil adsorption, substantial changes in surface elemental composition were detected (Table 3). Most notably, the relative atomic percentage of carbon increased markedly from 62.76% to 86.58%, while the signals corresponding to heteroatom-containing functional groups decreased significantly. The contents of N 1s and O 1s decreased from 5.15% to 0.59% and from 24.77% to 9.85%, respectively. Similarly, the P 2p signal decreased dramatically from 2.86% to 0.25%, whereas Na 1s became nearly undetectable after adsorption. Considering that XPS mainly probes the outermost surface layer (∼10 nm), these compositional changes strongly suggest that the composite surface became covered by hydrocarbon-rich species derived from the absorbed oil phase, thereby partially masking the underlying polar functionalities of the HH/CS matrix.

Interestingly, trace Zn 2p_3/2_ (0.13 at%) and increased Ca 2p content were detected after adsorption, which could be attributed to lubricant additives commonly present in commercial engine oils, such as zinc-containing antiwear agents and calcium-based detergent/dispersant compounds. These findings further support the successful confinement and retention of oil-derived components within the porous composite framework after sorption. Due to the attenuation effect caused by the adsorbed oil overlayer, the high-resolution N 1s and S 2p spectra after adsorption exhibited relatively weak signal intensity and are therefore provided in the SI (Fig. S4). Detailed binding energy assignments for the deconvoluted XPS components are summarized in Table S4 (SI).

The deconvoluted high-resolution C 1s spectrum of pristine HH/CS-2.0 (Fig. 10c) could be resolved into several characteristic components, including the dominant C–C/C–H peak centered at 284.8 eV, accompanied by contributions attributed to C–N/C–O and amide/carbonyl-related species at higher binding energies.^56^ A minor low-binding-energy component detected near ∼283.0 eV may be associated with defect-related or amorphous carbonaceous environments within the biopolymer matrix. After oil adsorption (Fig. 10g), the C–C/C–H component became substantially more dominant, indicating enrichment of aliphatic hydrocarbon species on the composite surface following oil uptake. In addition, the appearance of a weak low-energy carbon component near ∼283.9 eV may be associated with carbonaceous residues originating from degraded lubricant fractions.

Similarly, the O 1s spectrum before adsorption (Fig. 10d) mainly consisted of oxygen-containing functionalities associated with hydroxyl, carbonyl, amide, and phosphate-related groups originating from CS, keratin, and TPP crosslinking structures.^57^ After adsorption (Fig. 10h), the overall oxygen signal intensity decreased considerably, suggesting partial shielding of hydrophilic functional groups by the adsorbed oil layer. Slight variations in the O 1s envelope were also observed after sorption, which may be associated with oxygen-containing species present in oxidized lubricant components and additive residues retained within the porous network.

Furthermore, deconvolution of the high-resolution N 1s and S 2p profiles prior to sorption provided additional evidence regarding the hybrid network structure. The N 1s region exhibited a component near ∼402.1 eV assigned to protonated –NH_3_^+^ groups, supporting the successful ionic crosslinking interaction between protonated CS chains and TPP anions.^58^ Combined with the homogeneous distributions of P and Na revealed by SEM-EDX mapping and the quantitative XPS elemental analysis (Table 3), these results collectively provide strong evidence for the successful formation of the TPP-ionically crosslinked HH/CS network. In the S 2p spectrum, the characteristic disulfide-related sulfur species (S–S) originating from keratin proteins remained observable, confirming the preservation of the keratin structure after composite fabrication. These findings indicate that the chemical functionalities of the HH/CS composite remained essentially unchanged after oil sorption. The preserved keratin- and CS-related functional groups mainly maintained the structural framework of the sorbent, whereas oil uptake was governed predominantly by capillary infiltration, physical confinement within the interconnected pores, and weak hydrophobic interactions, rather than irreversible chemical reactions.

**Fig. 11:**
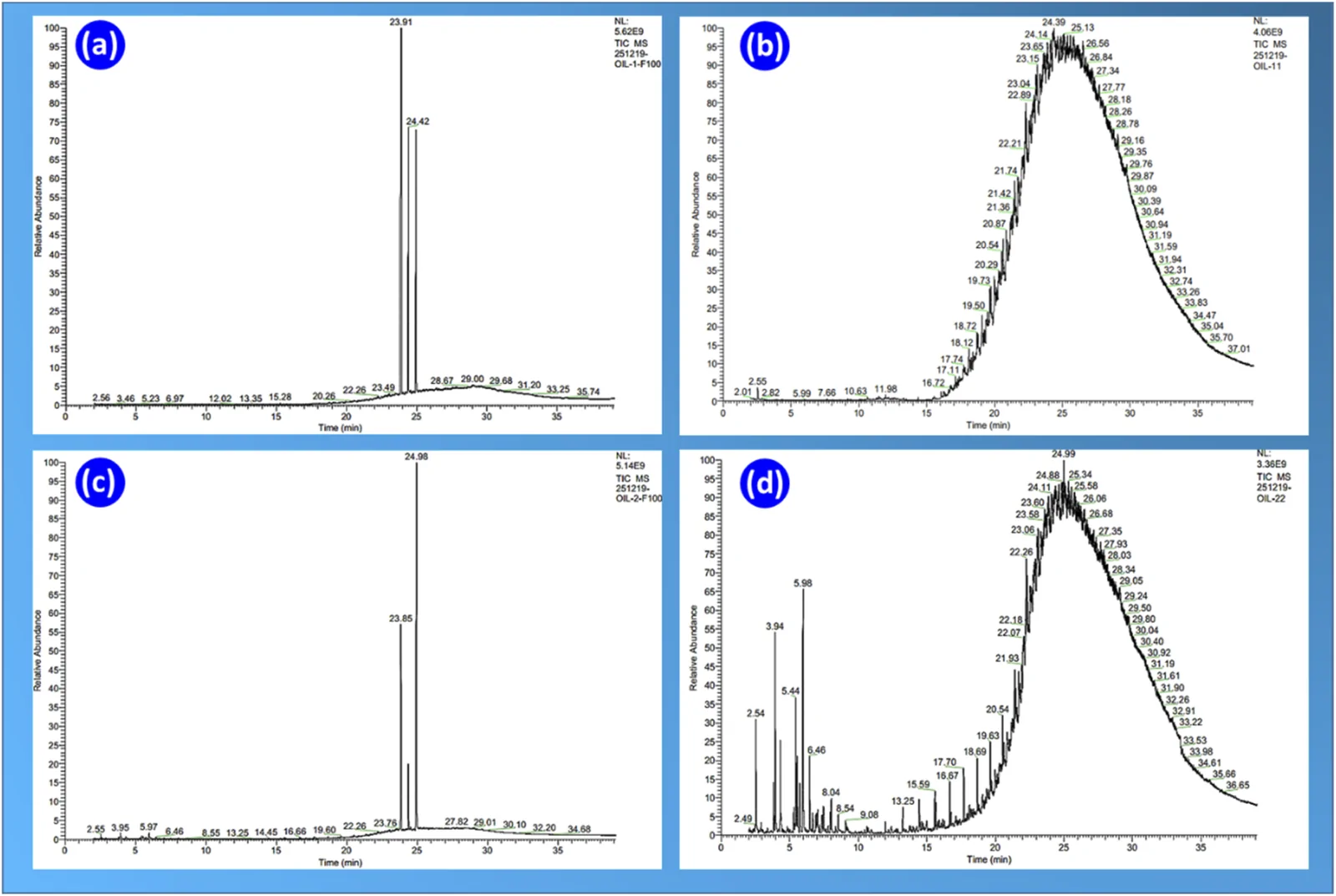
Total ion chromatograms (TIC) obtained from GC-MS analysis of (a) fresh engine oil, (b) used engine oil, (c) fresh engine oil recovered from the oil-loaded HH/CS composite mat after *n*-hexane extraction, and (d) used engine oil recovered from the oil-loaded HH/CS composite mat after *n*-hexane extraction.

To further elucidate the oil sorption behavior and evaluate possible compositional alterations during the adsorption–desorption process, GC-MS analysis was performed on both the original oils and the corresponding fractions recovered from the oil-loaded HH/CS composite mats after *n*-hexane extraction. The total ion chromatograms (TIC) are presented in Fig. 11.

As shown in Fig. 11a, the fresh engine oil exhibited a broad unresolved chromatographic envelope, characteristic of complex mixtures composed predominantly of linear and branched aliphatic hydrocarbons. After adsorption and solvent extraction from the HH/CS composite mat (Fig. 11c), the recovered oil displayed a highly similar TIC profile to that of the original fresh oil, with no noticeable disappearance of major chromatographic regions or formation of new peaks. This observation indicates that the adsorption process did not induce significant chemical transformation or selective fractionation of the hydrocarbon components. Instead, oil uptake mainly proceeded through physical confinement mechanisms, including capillary infiltration and hydrophobic interactions within the porous network of the composite mat.

A comparable trend was observed for the used engine oil samples. Compared with fresh oil, the TIC profile of the used oil (Fig. 11b) exhibited a broader and more irregular chromatographic distribution extending toward longer retention times, reflecting the presence of oxidized hydrocarbons, degraded lubricant fractions, and heavier molecular species generated during engine operation. A similar unresolved chromatographic hump has also been reported for waste lubricating oils in previous studies, where the broadened TIC baseline was associated with the accumulation of oxidized hydrocarbons, polycyclic aromatic hydrocarbons (PAHs), combustion-derived residues, and other high-molecular-weight degradation products formed during engine operation. Such chromatographic behavior is widely recognized as a characteristic fingerprint of chemically aged and thermally degraded lubricating oils.^59,60^ Notably, the chromatogram of the recovered used oil after adsorption–desorption treatment (Fig. 11d) retained nearly the same overall chromatographic characteristics as the original used oil. This result suggests that the HH/CS mat effectively trapped complex hydrocarbon mixtures without causing substantial molecular degradation during the sorption process.

Thus, the GC-MS results, together with the FTIR and XPS analyses, provide strong evidence that oil sorption in the HH/CS composite mat is predominantly governed by physical adsorption mechanisms rather than irreversible chemical reactions. The interconnected porous architecture of the hybrid matrix enables efficient confinement and recovery of both fresh and degraded lubricating oils while preserving the fundamental chemical composition of the adsorbed hydrocarbons.

### 3.6. Reusability and structural stability

**Fig. 12:**
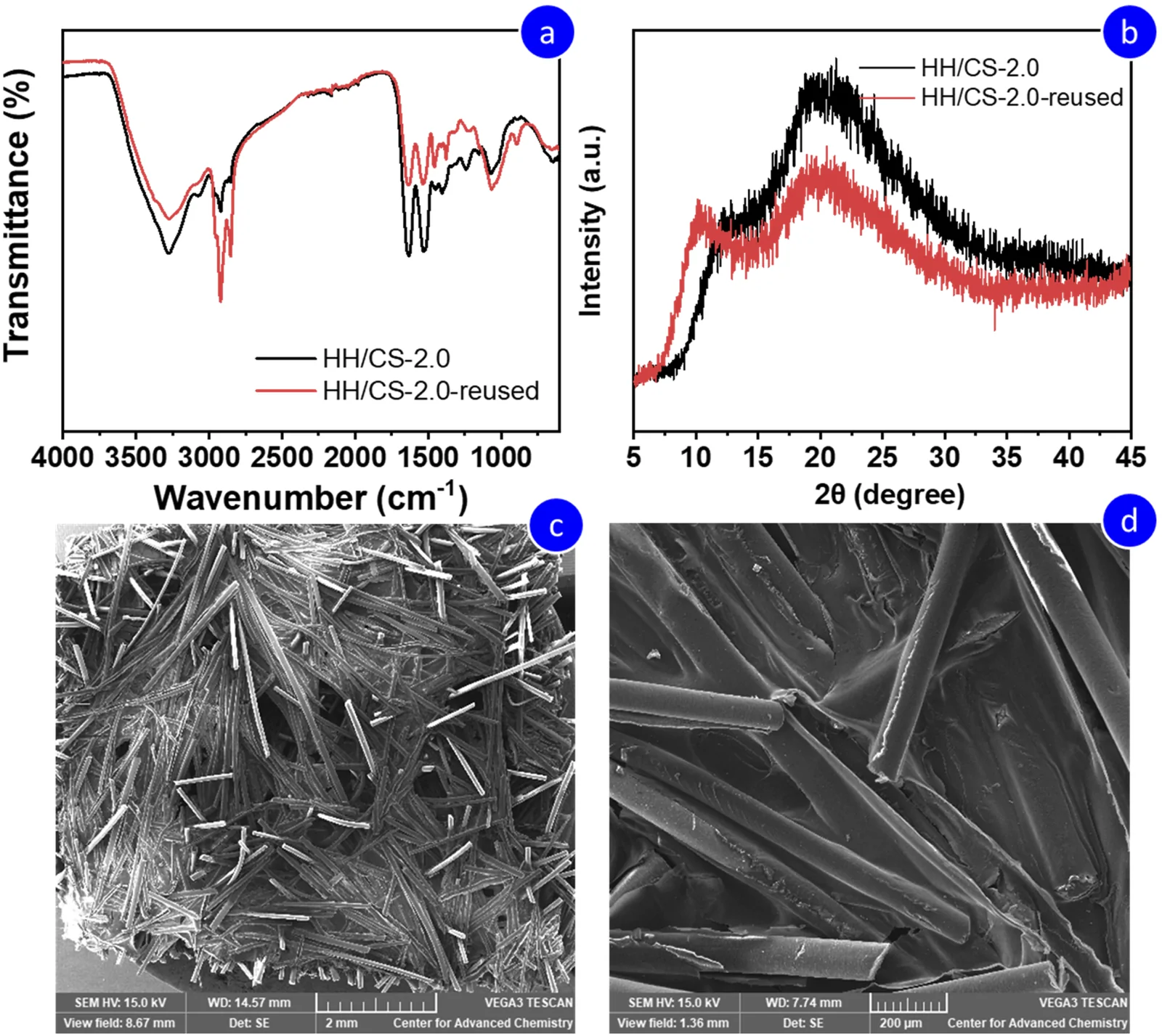
FTIR, XRD, and SEM analyses of the HH/CS-2.0 composite mat before and after five adsorption–desorption cycles in seawater using fresh engine oil: (a) FTIR spectra, (b) XRD patterns, and SEM images of the reused mat at (c) low and (d) high magnification.

To further evaluate the potential applicability of the HH/CS-2.0 composite mat for repeated oil recovery operations, the structural stability of the material after multiple adsorption–desorption cycles was investigated using FTIR, XRD, and SEM analyses (Fig. 12). As discussed in the previous sections, the composite mat retained considerable oil sorption capacity after five reuse cycles under both distilled water and seawater conditions, indicating promising regeneration performance and mechanical durability.

The FTIR spectra before and after five adsorption–desorption cycles (Fig. 12a) exhibited largely preserved characteristic absorption bands associated with keratin and CS structures. The broad O–H/N–H stretching region and the amide-related bands remained detectable after repeated reuse, suggesting that the fundamental chemical backbone of the hybrid matrix was maintained during cyclic operation. Meanwhile, the persistence of relatively strong aliphatic C–H stretching bands near 2920–2850 cm^−1^ in the reused sample may be attributed to residual hydrocarbon species retained within the porous framework after repeated oil exposure. Importantly, no significant emergence of new bands or disappearance of major functional groups was observed, indicating that the adsorption–desorption process did not induce substantial chemical degradation of the composite structure.

The XRD patterns of the pristine and reused HH/CS-2.0 mats are presented in Fig. 12b. Both samples exhibited a broad diffraction hump characteristic of predominantly amorphous biopolymer networks. After repeated reuse cycles, the overall diffraction profile remained largely unchanged, although slight variations in peak intensity and baseline broadening were observed, possibly due to residual oil deposition and minor rearrangement of polymer chains during cyclic swelling and drying processes. The absence of major crystalline collapse or structural transformation further confirms the robust structural stability of the hybrid mat during repeated sorption operations.

The SEM images of the reused HH/CS-2.0 mat after five cycles in seawater are shown in Fig. 12c and d. Even after repeated adsorption–desorption treatments, the composite maintained its interconnected three-dimensional fibrous architecture without severe structural collapse or fragmentation. The embedded HH fibers remained well integrated within the CS matrix, while the rough hierarchical surface morphology and interconnected void channels were still clearly observable. At higher magnification (Fig. 12d), partial surface coating and localized aggregation could be observed on several fiber surfaces, likely resulting from residual viscous hydrocarbon deposits remaining after repeated oil sorption cycles. Nevertheless, the overall porous framework and interfacial integrity of the composite were effectively preserved.

Overall, the combined FTIR, XRD, and SEM results demonstrate that the HH/CS-2.0 composite mat possesses excellent structural robustness and physicochemical stability during repeated adsorption–desorption operations. The preservation of the interconnected porous architecture together with the minimal chemical and morphological deterioration indicates that the developed hybrid mat can withstand repeated regeneration without significant structural degradation. These observations are consistent with the stable TPP-mediated ionic crosslinked network, which effectively maintains the integration of the keratin fibers within the CS matrix during repeated solvent extraction and drying. In addition, the relatively low mass loss (<9%) observed after five regeneration cycles further suggests that the composite framework possesses good resistance against fiber detachment and structural deterioration during repeated use. At the end of its service life, when progressive structural deterioration or diminished sorption capacity renders further regeneration ineffective, the spent HH/CS composite mats should be collected and managed as oil-contaminated solid waste in accordance with local environmental regulations. Although the composite is predominantly composed of bio-based HH and CS, the retained hydrocarbons preclude uncontrolled environmental disposal or natural degradation after use. Instead, controlled thermal treatment with energy recovery (incineration) represents a practical and sustainable end-of-life management strategy, enabling safe destruction of the adsorbed hydrocarbons while recovering energy from the bio-based matrix.

It should be noted, however, that the present evaluation was performed under controlled static laboratory conditions. In practical oil spill scenarios, dynamic environmental factors such as wave action, continuous water flow, fluctuating temperatures, and long-term environmental exposure may influence the sorption behavior and structural durability of the composite mats. Therefore, future studies will focus on evaluating the HH/CS sorbents under dynamic aquatic systems and pilot-scale conditions to further validate their practical applicability.

## 4. Conclusions

In summary, a highly porous, sustainable, and reusable bio-based oil sorbent was successfully fabricated by integrating waste HH fibers into a CS matrix *via* a facile sodium tripolyphosphate (TPP)-mediated ionic crosslinking strategy. The optimized HH/CS-2.0 composite mat possessed a robust interconnected hierarchical framework, yielding a high maximum sorption capacity of 4864.46 mg g^−1^ toward motorcycle engine oil under optimized conditions. The oil sorption performance was strongly influenced by operational parameters, where lower temperatures enhanced capillary-driven infiltration due to increased fluid viscosity, while greater mat thickness expanded the accessible pore volume and oil-retention capability. The engineered composite mats demonstrated effective remediation performance for both fresh and used engine oils at simulated oil–water interfaces. Although the presence of seawater slightly reduced the initial sorption capacity because of salinity-induced interfacial effects, the HH/CS mats maintained excellent structural durability and regeneration capability, exhibiting relatively stable oil-retention performance over five consecutive sorption–desorption cycles. A systematic combination of FTIR, XPS, and GC-MS analyses provided strong evidence that oil uptake was predominantly governed by physical sorption mechanisms, including hydrophobic interactions, surface wetting, capillary-driven infiltration, and physical confinement within the interconnected porous architecture. Moreover, no significant chemical transformation or selective fractionation of the captured hydrocarbon components was observed during the sorption–desorption process. Complementary FTIR, XRD, and SEM analyses further confirmed the structural and chemical stability of the composite framework after repeated reuse cycles. This study demonstrates a green, low-cost, and efficient strategy for converting biopolymer wastes into high-performance sorbent materials for potential oil spill cleanup and oily wastewater remediation applications.

## Author contributions

Van Dat Doan: writing – original draft, investigation, formal analysis, conceptualization. Thi Kim Thuy Nguyen: investigation, formal analysis. Vy Anh Tran: data curation, formal analysis. Ngoc Nga Ho: investigation, conceptualization. Hien Y. Hoang: investigation, formal analysis, methodology. Quoc Bao Tran: writing – review & editing, formal analysis, data curation, conceptualization. Van Thuan Le: writing – review & editing, supervision, methodology, data curation, conceptualization.

## Conflicts of interest

The authors declare that they have no known competing financial interests or personal relationships that could have appeared to influence the work reported in this paper.

## Supplementary Material

RA-016-D6RA04911G-s001

## Data Availability

The data that support the findings of this study are available from the corresponding author upon reasonable request. Supplementary information (SI) is available. See DOI: https://doi.org/10.1039/d6ra04911g.
