# Supplementary material for: Hierarchically porous waste human hair/chitosan composite mats enabling reusable capture of fresh and spent motor oils from freshwater and marine environments
Source: RSC Adv. 2026 Aug 12;16(40):47108–32. doi: 10.1039/d6ra04911g (PMC13463122; doi:10.1039/d6ra04911g)
Supplement: Supplementary file 1 [file RA-016-D6RA04911G-s001.pdf]

Supplementary Information (SI) for RSC Advances.
This journal is © The Royal Society of Chemistry 2026

Hierarchically Porous Waste Human Hair/Chitosan Composite Mats Enabling Reusable Capture of Fresh and Spent Motor Oils from Freshwater and Marine Environments

Van Dat Doan[a], Thi Kim Thuy Nguyen[b], Vy Anh Tran[c], Ngoc Nga Ho[a], Hien Y Hoang[b,d], Quoc Bao Tran[e,*], Van Thuan Le[b,d,*]

[e]*Faculty of Chemical Engineering, Industrial University of Ho Chi Minh City, Ho Chi Minh City, 700000, Viet Nam*

[b]*Faculty of Environmental and Natural Sciences, Duy Tan University, 03 Quang Trung, Da Nang City, 550000, Viet Nam.*

[c]*Faculty of Applied Science and Technology, Nguyen Tat Thanh University, Ho Chi Minh City, 700000, Viet Nam*

[d] *Center for Advanced Chemistry, Institute of Research & Development, Duy Tan University, 03 Quang Trung, Da Nang City, 550000, Viet Nam*

[e]*Faculty of Environment, Ho Chi Minh City University of Natural Resources and Environment, Ho Chi Minh City, 70000, Viet Nam*

**Corresponding authors:*
Quoc Bao Tran (tqbao@hcmunre.edu.vn)

Van Thuan Le (levanthuan3@duytan.edu.vn)

Table S1. Physical characteristics of HH/CS composite mats prepared at different mass ratios. Values are presented as mean ± standard deviation (n = 6).

HH/CS ratio	Diameter (mm)	Thickness (mm)	Mass (g)
HH/CS-1.0	38.93 ± 0.69	2.14 ± 0.51	0.7825 ± 0.009
HH/CS-1.5	40.60 ± 1.21	2.24 ± 0.18	0.9740 ± 0.008
HH/CS-2.0	43.33 ± 0.59	2.69 ± 0.62	1.2130 ± 0.017
HH/CS-2.5	45.04 ± 0.71	3.67 ± 0.38	1.4105 ± 0.005
HH/CS-3.0	45.31 ± 0.34	4.60 ± 0.53	1.6340 ± 0.072

Determination of bulk density and porosity

The bulk density and porosity of the HH/CS composite mats were determined using a geometrical method. Briefly, the composite mats were cut into square specimens with dimensions of approximately 10 × 10 mm. The length, width, and thickness of each sample were measured using a digital caliper, and the sample mass was determined using an analytical balance.

The bulk density (ρ_{bulk}) of the composite mats was calculated according to the following equation:

$$\rho_{bulk=} \frac{m}{V}$$

where m is the mass of the sample (g) and V is the apparent volume of the sample (cm^3), calculated from the measured dimensions.

The theoretical solid density (ρ_{solid}) of each composite mat was estimated based on the mass fractions and intrinsic densities of the individual components using the rule of mixtures:

$$\rho_{solid} = \frac{1}{\left(\frac{w_{HH}}{\rho_{HH}} + \frac{w_{CS}}{\rho_{CS}}\right)}$$

where w_{HH} and w_{CS} are the mass fractions of human hair and chitosan, respectively, while ρ_{HH} and ρ_{CS} represent their corresponding intrinsic densities. The density of

human hair and chitosan were taken as 1.32 and 1.60 g cm^{-3}, respectively, based on literature values (https://doi.org/10.1117/1.3184444; https://doi.org/10.3390/ma11112144).

The porosity (ε) of the composite mats was subsequently calculated using the following equation:

$$\varepsilon(\%) = \left(1 - \frac{\rho_{bulk}}{\rho_{solid}}\right) x\ 100$$

All measurements were conducted in triplicate, and the results are reported as mean ± standard deviation (**Table S2**).

Table S2. Bulk density, theoretical solid density, and calculated apparent porosity of HH/CS composite mats at different mass ratios

HH/CS ratio	ρ_{bulk} (g cm^{-3})	ρ_{solid} (g cm^{-3})	Porosity (%)
HH/CS-1.0	0.263 ± 0.030	1.446	81.8 ± 2.1
HH/CS-1.5	0.262 ± 0.022	1.419	81.5 ± 1.6
HH/CS-2.0	0.302 ± 0.042	1.402	78.5 ± 3.0
HH/CS-2.5	0.277 ± 0.008	1.389	80.1 ± 0.6
HH/CS-3.0	0.273 ± 0.008	1.380	80.2 ± 0.6

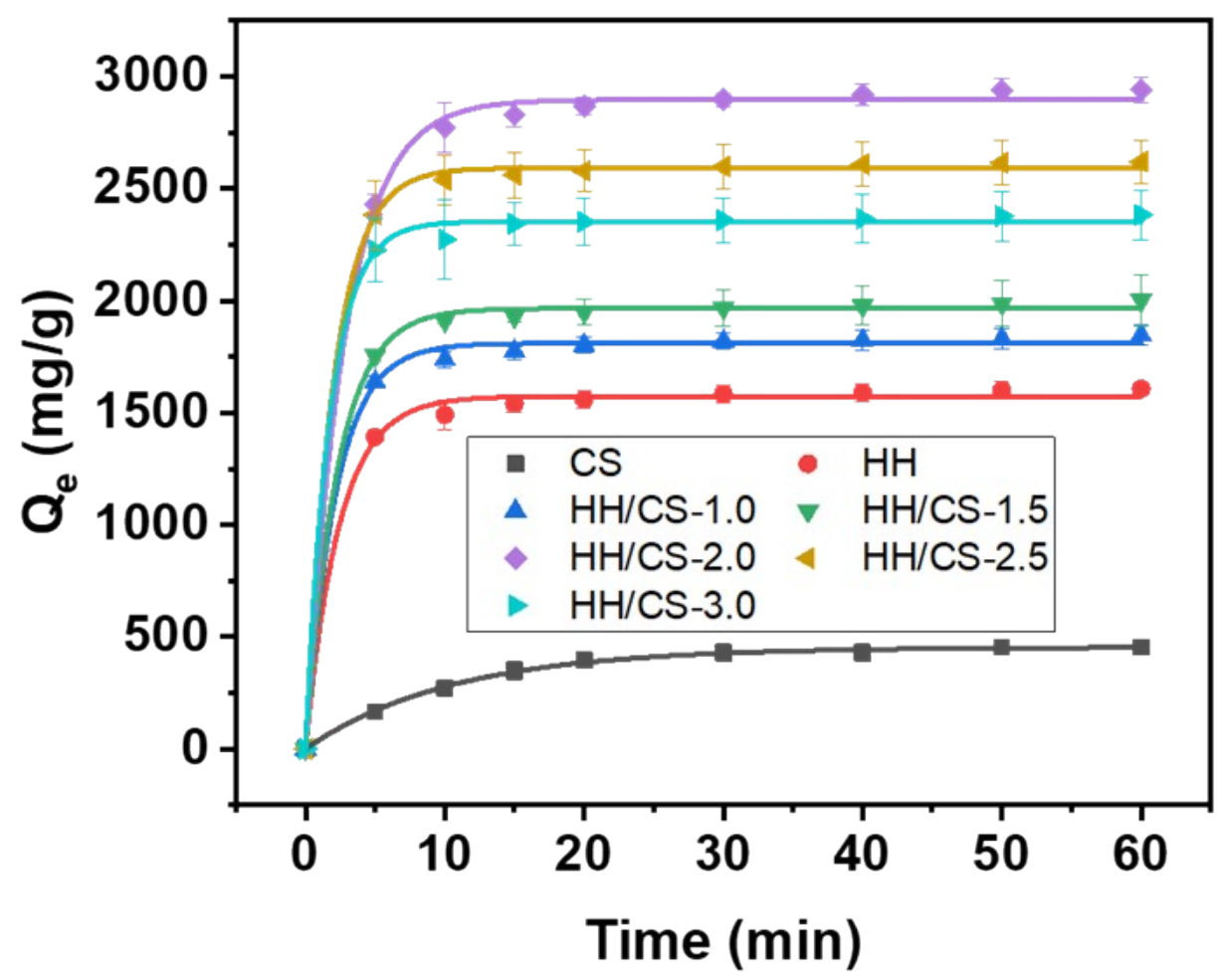

Fig. S1. Nonlinear pseudo-first-order kinetic fitting of the oil sorption data for CS, HH, and HH/CS composite mats

Table S3. Kinetic parameters obtained by fitting the oil sorption data of CS, HH, and HH/CS composite mats using pseudo-first-order (PFO) and pseudo-second-order (PSO) kinetic models.

Sample	Pseudo-first-order (PFO) $q_t = q_e(1 - e^{-k1t})$				Pseudo-second-order (PSO) $q_t = (k_2 q_e^2 t)/(1 + k_2 q_e t)$			
	qe (mg g^{-1})	k_1 (min^{-1})	R^2	χ^2	q_e (mg g^{-1})	k_2 (g mg^{-1} min^{-1})	R^2	χ^2
**CS**	453.08 ± 4.80	0.0948 ± 0.0035	0.99766	64.65	540.92 ± 19.09	(1.996 ± 0.320) × 10^{-4}	0.98938	293.46
**HH**	1573.68 ± 12.55	0.4150 ± 0.0342	0.99672	1012.07	1627.24 ± 2.20	(7.142 ± 0.167) × 10^{-4}	0.99995	14.56
**HH/CS-1.0**	1809.55 ± 11.68	0.4587 ± 0.0350	0.99781	894.25	1859.45 ± 2.61	(7.898 ± 0.231) × 10^{-4}	0.99995	21.23
**HH/CS-1.5**	1965.90 ± 9.78	0.4381 ± 0.0242	0.99871	621.53	2021.40 ± 6.56	(6.848 ± 0.442) × 10^{-4}	0.99972	132.92
**HH/CS-2.0**	2897.53 ± 15.21	0.3564 ± 0.0163	0.99862	1435.44	3013.97 ± 17.72	(3.009 ± 0.253) × 10^{-4}	0.99914	896.50
**HH/CS-2.5**	2592.75 ± 9.22	0.4977 ± 0.0236	0.99933	564.56	2647.03 ± 5.90	(7.223 ± 0.419) × 10^{-4}	0.99987	112.06
**HH/CS-3.0**	2353.36 ± 12.19	0.5703 ± 0.0500	0.99856	1003.33	2394.09 ± 6.90	(1.040 ± 0.099) × 10^{-3}	0.99977	157.09

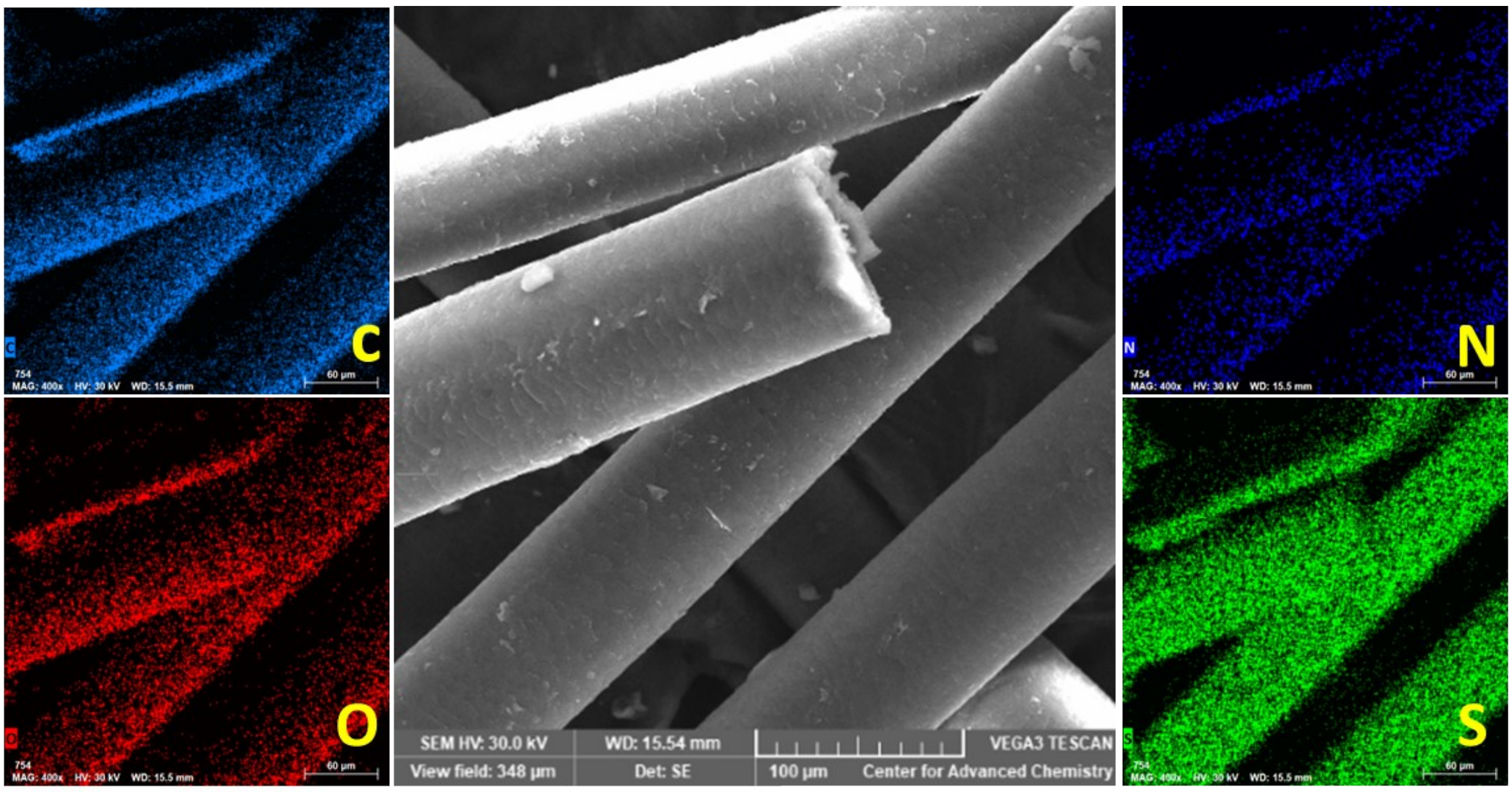

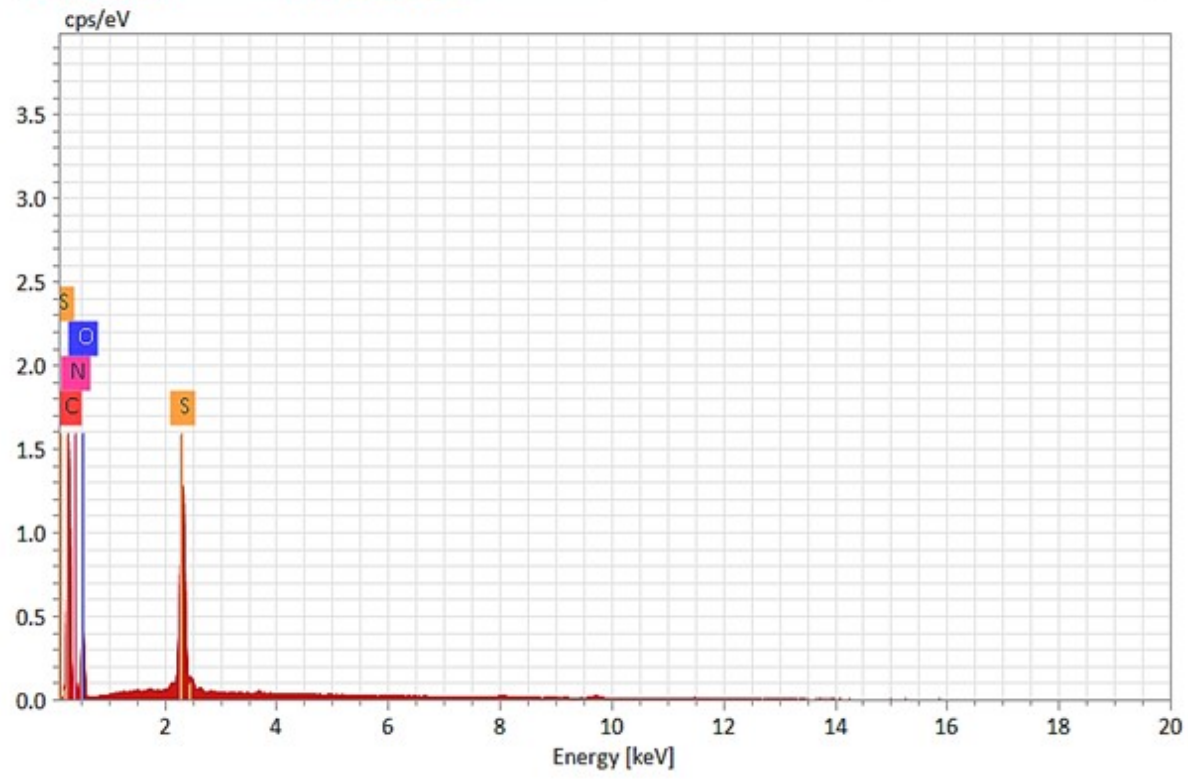

Element	Wt. %	At. %
C	53.92	60.95
O	26.55	22.53
N	15.10	14.64
S	4.44	1.88
Total	100.00	100.00

Fig. S2. SEM image, elemental mapping, EDX spectrum, and elemental composition analysis of pristine HH fibers

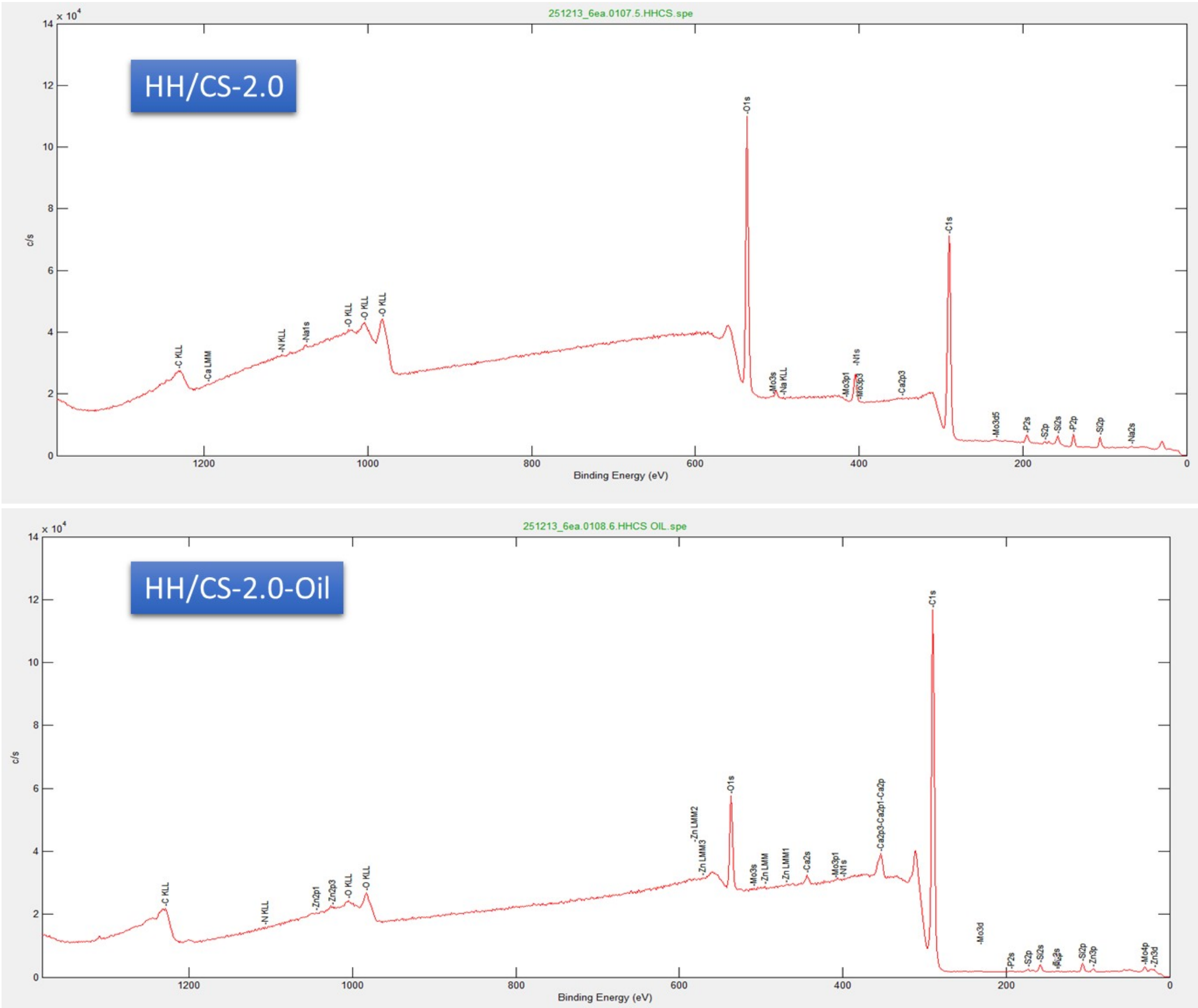

Fig. S3. Fully annotated wide-scan XPS survey spectra of the HH/CS-2.0 composite mat before and after engine oil sorption, detailing all auxiliary and trace elemental transitions.

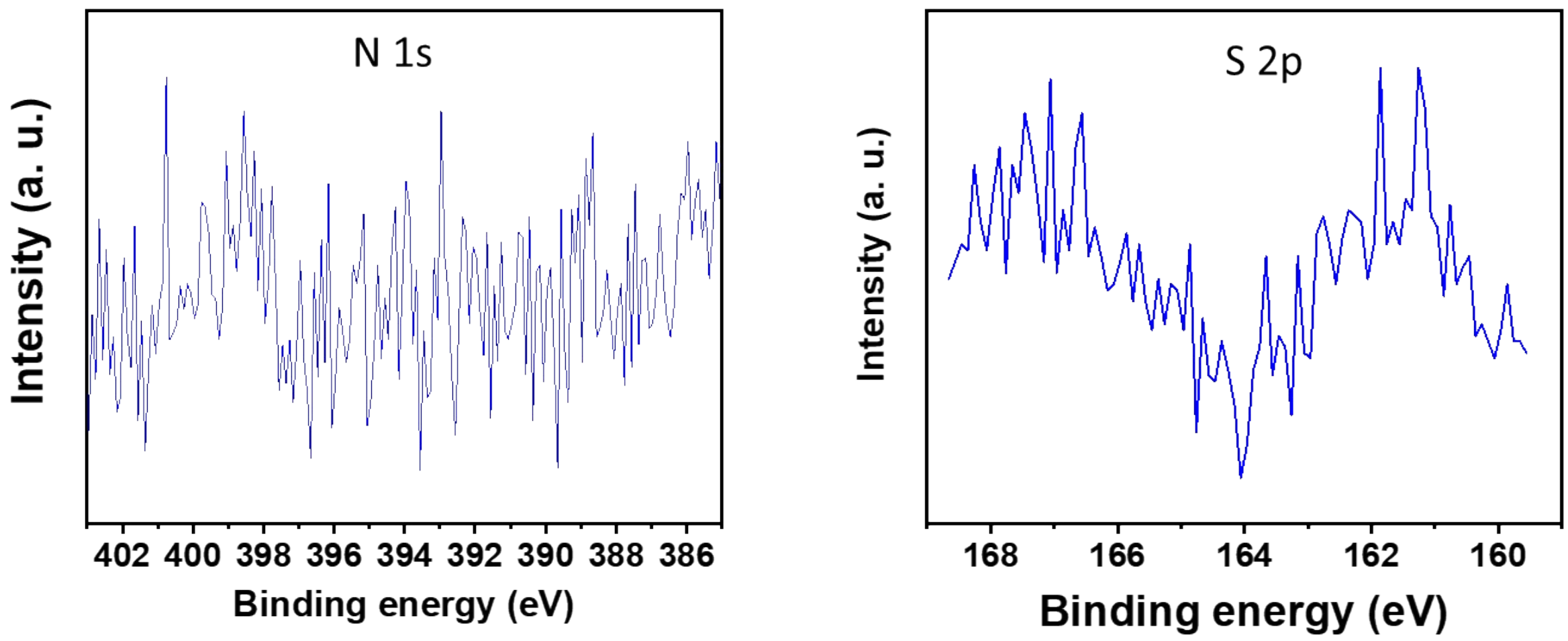

Fig. S4. High-resolution core-level XPS spectra of N 1s and S 2p of the HH/CS-2.0 composite mat after engine oil sorption

Table S4. Binding energy assignments of deconvoluted high-resolution XPS spectra of HH/CS-2.0 composite mats before and after oil sorption

Spectrum	Binding energy (eV)	Assignment
**Before sorption**		
C 1s	~284.8	C–C / C–H (aliphatic and aromatic carbon)
C 1s	~286.3	C–N / C–O functional groups
C 1s	~287.9	C=O / O–C=O (amide and carbonyl species)
C 1s	~283.0	Defect-related or low-coordination carbon species
O 1s	~531.5	C=O and P=O oxygen species
O 1s	~532.8	C–O / P–O groups
O 1s	~533.4	O–C–O or adsorbed oxygen species
O 1s	~529.7	Trace inorganic oxygen species
N 1s	~398.0	C=N or imine-type nitrogen
N 1s	~400.3	$–NH_2$ / amide nitrogen
N 1s	~402.1	Protonated $–NH_3^+$ groups
S 2p	~161.9	Reduced sulfur species
S 2p	~163.1	S–S / thiol-related sulfur
S 2p	~167.0	Sulfoxide/sulfone species
S 2p	~168.2	Oxidized sulfur species
**After sorption**		
C 1s	~284.8	C–C / C–H from hydrocarbon chains
C 1s	~283.9	Carbonaceous hydrocarbon residues
C 1s	~285.8	C–O / C–N species
C 1s	~288.8	O–C=O / oxidized carbon species
O 1s	~531.4	Carbonyl oxygen species
O 1s	~532.1	C–O / hydroxyl oxygen
O 1s	~530.6	Oxygen associated with inorganic additive residues
O 1s	~529.7	Trace inorganic oxygen species
